# Functional Hybrid Molecules for the Visualization of Cancer: PESIN‐Homodimers Combined with Multimodal Molecular Imaging Probes for Positron Emission Tomography and Optical Imaging: Suited for Tracking of GRPR‐Positive Malignant Tissue**

**DOI:** 10.1002/chem.202002386

**Published:** 2020-10-28

**Authors:** Ralph Hübner, Xia Cheng, Björn Wängler, Carmen Wängler

**Affiliations:** ^1^ Biomedical Chemistry, Department of Clinical Radiology and Nuclear Medicine Medical Faculty Mannheim of Heidelberg University Theodor-Kutzer-Ufer 1–3 68167 Mannheim Germany; ^2^ Molecular Imaging and Radiochemistry Department of Clinical Radiology and Nuclear Medicine Medical Faculty Mannheim of Heidelberg University Theodor-Kutzer-Ufer 1–3 68167 Mannheim Germany

**Keywords:** fluorescent dyes, multimodal imaging, optical imaging, PESIN homodimers, positron emission tomography

## Abstract

We describe multimodal imaging probes for gastrin‐releasing peptide receptor (GRPR)‐specific targeting suited for positron emission tomography and optical imaging (PET/OI), consisting of PESIN (PEG_3_‐BBN_7‐14_) dimers connected to multimodal imaging subunits. These multimodal agents comprise a fluorescent dye for OI and the chelator ((1,4,7‐triazacyclononane‐4,7‐diyl)diacetic acid‐1‐glutaric acid) (NODA‐GA) for PET radiometal isotope labelling. Special focus was put on the influence of the used dyes on the properties of the whole bioconjugates. For this, several compounds with different fluorescent dyes and non‐dye carrying subunits were synthesized and investigated. As fluorescent dyes, dansyl, NBD, derivatives of fluorescein, coumarin and rhodamine as well as three pyrilium‐based dyes were employed. Considerable influence of the charge of the colored unit on hydrophilicity as well as in vitro target receptor binding was observed and classified. High radiochemical yields and purities were found during radiolabeling of the multimodal imaging subunits as well as their GRPR‐specific bioconjugates with ^68^Ga. Examinations of the photophysical properties of both molecule species displayed no loss or alteration of fluorescence characteristics.

## Graphical abstract

**Multimodal imaging**: Different PESIN‐homodimers, suited for positron emission tomography and optical imaging, were synthesized. Special focus was set on the influence of different dyes on biomedical‐related properties.

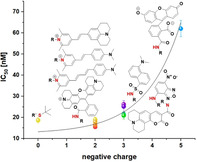

## Introduction

Multimodal imaging, based on molecular probes, becomes an important and growing field in medical diagnostics.^[1]^ Imaging, characterization and staging of cancer and other diseases can be more reliable and sustainable with suited modalities and enhance the quality of diagnosis of physiological and pathogen conditions. For multimodal imaging employing two modalities, combinations of several complementary technologies are conceivable. Besides PET and OI, X‐ray‐based computed tomography (CT), magnetic resonance imaging (MRI), photoacoustic ultrasonic (US) imaging and single photon emission computed tomography (SPECT) are used on a daily basis as imaging techniques in clinical routine.^[1a]^ In clinical nuclear medicine, e.g., the combination of PET and SPECT with CT is commonly applied since the early 2000s.^[2]^ PET/MRI, enabling functional imaging and the corresponding morphological correlation with much higher soft tissue contrast than PET/CT emerges more and more important in clinical application since 2010.[^1a^, ^3^] Other combinations of imaging methodologies which currently are in the focus of intensive investigations are for example, MRI/OI,^[3b]^ US/OI^[4]^ and PET/OI.^[5]^

Each modality brings its own specific advantages and can be sorted in one of two subgroups:^[6]^ One consists of those imaging techniques enabling a high sensitivity (based on the amount of contrast agent used) and thus target‐specific functional imaging but providing only minimal morphological information. PET, SPECT and OI are typical representatives of this group. The other group comprises those techniques which provide rich and detailed morphological information such as MRI, US and CT. These however exhibit a comparably much lower imaging sensitivity requiring high amounts of contrast agents, precluding functional imaging. To obtain best imaging results, modalities of both groups are combined. These combinations thus enable to gather complementary information simultaneously in a short time period, drastically improving medical diagnosis.

At first glance, the PET/OI combination does not seem to offer such favorable complementary information as both are found in the imaging subgroup of “high sensitivity and little morphological information provided”.[^5d^, ^6^] In principle, PET offers a slightly higher sensitivity than OI, enabling a target visualization at a very low (pmol) amount of applied radiolabeled substance whereas OI necessitates substance quantities of nmol to pmol.^[7]^ Considering, however, that nmolar amounts are usually used for the radiolabeling reaction, resulting in the required pmol amounts of radiolabeled substance and leaving the rest of the nmolar precursor in unlabeled form (being however also applied to the human subject), the overall quantity of multimodal imaging agent applied remains the same and is adequately detectable by both imaging modalities.

Furthermore, the combination of PET/OI features certain important advantages over other imaging techniques. In fact, PET/OI offers the possibility of performing pre‐ and intra‐operative imaging.[^5a^, ^5b^] PET on the one hand shows extraordinary benefits in pre‐operative, whole‐body imaging as the gold‐standard [^18^F]FDG and other PET nuclide‐labeled radiotracers demonstrate daily.^[8]^ In contrast, optical imaging, based on the emission of fluorescent light, is seldomly found in clinical application but holds a great promise in medical imaging. For this purpose, fluorescent dyes like indocyanine green (ICG) have been used over the last 60 years.^[9]^ Fluorescence‐based OI is discussed and under development for example, for image‐guided surgery^[10]^ together with lymph node mapping.[^1d^, ^10c^] Spectroscopically fluorescence‐guided surgery offers the advantage of real time imaging during surgical intervention. The surgeon can benefit for example, from directly distinguishing between malignant and non‐malignant tissue.^[11]^ The precise removal of cancerous tissue and malignantly transformed lymph nodes minimizes tumor recurrence and maximizes the speed of recovery of patients and can dramatically improve the prognosis for the patient. Additionally, fluorescence‐supported, optically‐guided biopsies, even under robotic assistance, are in the focus of current developments. The precise distinction between target and non‐target tissue goes together with additional information which can be obtained by this technique such as tumor heterogeneity and histological characterization by visualization of biomarkers expressed on the cell surface. Not least mentioning photothermal or photodynamic effects^[12]^ on which photo‐theranostic applications are based on.^[13]^

To be able to obtain optimal imaging results by PET/OI, suitable combined imaging agents have to be available. Such multimodal agents possess the advantage of co‐localization of both signaling units as the use of a single probe results in the same pharmacokinetics for both the fluorescent dye and the radioactive nuclide.^[1a]^ In addition, the use of a combined probe minimizes the regulatory hurdles for human use compared to bringing two different imaging agents (that have furthermore to be optimized to enable comparable imaging results in PET and OI) into clinical application. Such combined imaging agents to be used in PET/OI have to exhibit a smart design to meet the requirements for such probes to be visualized by different imaging modalities and to avoid physical and chemical interference between the molecular functions. For instance, a dye unit with heteroatom donors could strongly affect radiolabeling with positron‐emitters or the positron‐emitting nuclide chelator moiety could interact with the dye and result in decrease, shift or quenching of fluorescence emission characteristics.

Designing multimodal imaging agents for PET/OI, the different building blocks which need to be integrated to obtain a fluorescent, positron‐emitting and target tissue‐specific multimodal agent have to be chosen carefully:

One is the positron‐emitting nuclide to be applied as several β^+^‐emitters are suitable for medical imaging.^[14]^ Among other things to consider are the physical characteristics such as the energy of the emitted positron and thus the mean free path of the positron before annihilation and thus γ‐emission takes place, directly influencing the resolution of the PET images. Furthermore, the half‐life as well as the production mode (generator or cyclotron) of the respective nuclides and thus their broad accessibility are of importance. Equally important is of course the chemistry of the β^+^‐emitter being required for radiolabel introduction into the molecular probe. Due to the short half‐life of most diagnostic β^+^‐emitters and the common‐sense avoidance of every unnecessary radioactivity handling, the radiolabeling will always be the last synthesis step for preparing a multimodal PET/OI agent. Macrocyclic chelators and their well‐established coordination chemistry show the advantages to selectively trap metal ions fast and under mild conditions and thus have found widespread use in clinically relevant radiotracers. The macrocycle is usually introduced into the biomolecule used as targeting vector either via click chemistry or even more simple, by acid‐amide coupling.

Also, an appropriate targeting vector has to be chosen and adequately functionalized to be able to be used in the context of a multimodal imaging agent. For the target‐specific uptake of imaging agents into malignancies, certain receptors which are overexpressed on a specific tumor entity are in general addressed. This can be achieved by using receptor‐specific biomolecules and their analogs, serving as tumor‐targeting vectors. For this purpose, different biomolecule classes can be used: antibodies, specifically binding their antigen with very high affinity and specificity,^[15]^ small molecules such as acetylaspartylglutamate used for prostate‐specific membrane antigen (PSMA) binding^[11a]^ or vitamin B9 for folate receptor targeting^[16]^ and peptides such as somatostatin, vasopressin or bombesin for G protein‐coupled receptors (GPCRs).[^8a^, ^17^]

Finally, also the fluorescent dye to be used for OI during combined PET/OI and its conjugation chemistry have to be chosen. A huge library of dye molecules with very variable fluorescent characteristics exists.[^10d^, ^16^, ^18^] Many of them can be conjugated to biomolecules without losing the intended photophysical characteristics. Also the optical properties of the dye have to be taken into consideration, among them the absorption maximum, Stokes shift, emission maximum, extinction coefficient and quantum yield.

Noteworthy mentioning that different molecular functions in one single hybrid probe for multimodal medical imaging can interact with each other. In this study, we shed light on the fundamental question how different fluorescent dyes interact with a given chelator‐modified biomolecule, influencing each other's chemical, biological and photophysical properties. For our proof of principle study, we chose a homodimeric PESIN (PEG_3_‐BBN_7‐14_) backbone to address the gastrin‐releasing peptide receptor (GRPR) which is overexpressed on different cancers.[^9b^, ^18d^, ^19^] Although the endogenous ligand for the GRPR, the 27‐amino acid receptor ligand bombesin (BBN), exhibits a high affinity towards its target, it is also known to suffer from a low in vivo stability. Thus, different derivatives of BBN such as PESIN were developed, presenting with somewhat lower affinities, but considerably increased stabilities. In order to increase the affinity of BBN analogs to the GRPR, dendritic, particularly dimeric PESIN arrangements with more than one peptide copy were developed and shown to increase affinity, sensitivity and specificity towards the GRPR compared to their monomeric counterparts.^[20]^ In addition, the homodimerization of tumor‐targeting peptides can have a beneficial effect on ligand stability in general^[21]^ which was also shown for PESIN dimers before.^[20a]^ As positron‐emitter, we chose the clinically relevant nuclide ^68^Ga (t_1/2_=68 min), which can be readily obtained by a commercially available ^68^Ge/^68^Ga‐generator system and chemically be stably introduced by complexation under mild labeling condition (pH 4; 45 °C, 10 min) using the NODA‐GA ((1,4,7‐triazacyclononane‐4,7‐diyl)diacetic acid‐1‐glutaric acid) macrocycle.[^14a^, ^17^] The use of the NODA‐GA chelator offers the additional advantage of being able to stably complex a second positron‐emitting radiometal cation, namely ^64^Cu (t_1/2_=12.07 h), under comparably mild labeling conditions, leaving room for choosing the most adequate radionuclide for the respective in vivo application^[19i]^ which might be especially favorable in case of slow in vivo pharmacokinetics. As fluorescent dyes, we chose to introduce a small library of different dyes, covering a range of properties, including different molecular sizes, charges, absorption and fluorescence properties, Stokes shifts and chemical conjugation characteristics, and to study their influence on the chemical, biological and photophysical properties of the resulting multimodal imaging agents.

## Results and Discussion

To obtain the target multimodal imaging agents comprising the molecular building blocks specified before, we first developed a synthesis strategy aiming at the convergent synthesis of the very complex final products.

**Scheme 1:**
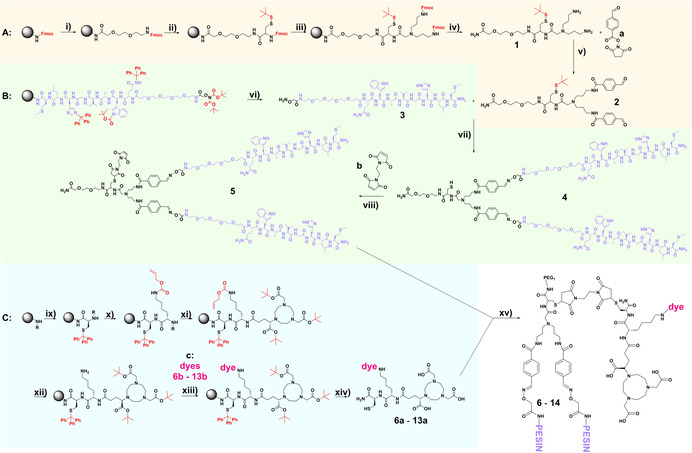
Schematic depiction of the synthesis of the maleimide‐modified PESIN‐dimer **5** (**A** and **B**) and its reaction with the multimodal imaging units (MIUs) **6 a**–**13 a** (**C**) to form the final hybrid multimodal imaging agents **6**–**13. A** (yellow background): Synthesis of the orthogonally functionalized branched dendron **2** (a: SFB=Succinimidyl‐*p*‐formyl‐benzoate). **B** (green background): Synthesis of aminooxy‐PESIN **3** and the maleimide‐modified PESIN‐dimer **5** (b: BME=1,2‐*bis*(maleimido)ethane). **C** (blue background): Synthesis of the multimodal imaging units (MIUs) **6 a**–**13 a**. (c: representative dye molecule). Reaction conditions: i) swelling 45 min; piperine/DMF (1:1), 5 min; 4 equiv. activated amino acid for 30 min (activation: 3.9 equiv. HBTU, 4 equiv. DIPEA, 2 min). ii) Piperine/DMF (1:1), 5 min; 4 equiv. activated amino acid for 30 min (activation: 3.9 equiv. HBTU, 4 equiv. DIPEA, 2 min). iii) Piperine/DMF (1:1), 5 min. iv) TFA/TIS (95:5 v/v); 60–120 min; HPLC. v) 2 equiv. SFB; pH 7.0–7.2; 30 min at 45 °C; HPLC. vi) TFA/TIS (95:5 v/v); 60–120 min; HPLC vii) a) 2.25 equiv. PESIN; pH 4.0–4.2; 60 min at 45 °C, b) 10 equiv. TCEP=(*tris*(2‐carboxyethyl)phosphine)); pH 4.0–4.2; 120–240 min at 45 °C; HPLC. viii) 3 equiv. BME; pH 7.0–7.2; 5 min; HPLC. ix) Piperine/DMF (1:1), 5 min; 4 equiv. activated amino acid for 30 min (activation: 3.9 equiv. HBTU, 4 equiv. DIPEA, 2 min). x) Piperine/DMF (1:1), 5 min; 4 equiv. activated amino acid for 30 min (activation: 3.9 equiv. HBTU, 4 equiv. DIPEA, 2 min). xi) Piperine/DMF (1:1), 5 min; 2 equiv. activated NODA‐GA(*tris*‐*t*Bu) for 60 min (activation: 1.95 equiv. HBTU, 2 equiv. DIPEA, 2 min). xii) 1.5 equiv. Pd(PPh_3_)_4_ and 10 equiv. morpholine in CH_2_Cl_2_ for 2 h under absence of light. xiii) 1.5 equiv. dye (different conditions depending on dye used—see experimental section for details). xiv) TFA/TIS (95:5 v/v); 60–120 min; HPLC. xv) 1.1 equiv. MIU; pH 7.0–7.2; 5 min; HPLC. As solvent for v, vii, viii, xv, a 1:1 MeCN:H_2_O + 0.1 % TFA mixture was used. SFB and BME were dissolved in MeCN + 0.1 % TFA, solvation of BME further assisted by ultrasonic bath at 50 °C for 5 min.

One building block to be synthesized was the PESIN peptide dimer serving as GRPR targeting vector which furthermore had to contain a functionality for chemoselective introduction of the multimodal imaging units (MIUs). The MIUs on the other hand should consist of the NODA‐GA chelator for ^68^Ga‐labeling, the fluorescent dye and a corresponding functional group for efficient conjugation to the peptide dimer targeting vector. This convergent synthesis strategy not only enables the efficient assembly of such complex, multifunctional constructs but also allows to introduce the synthesized MIUs into any appropriately functionalized biomolecule intended to be used for target‐specific accumulation. By this, it is possible to tailor multimodal imaging agents for a specific imaging purpose and tumor entity based on the MIUs described here. At first, we aimed at the synthesis of the tumor‐targeting vector, the appropriately functionalized, symmetrical PESIN‐dimer to which the MIUs can in the following be coupled efficiently and chemoselectively by click chemistry. The synthesis pathway to this molecule (**5**) is shown in Scheme 1 A,B. **5** was itself composed of different building blocks, the trifunctional linker **2** (PEG_1_‐*d*‐Cys(S‐S‐*t*Bu)‐TrL(SFB)_2_) and aminooxy‐modified PESIN **3** (H_2_N‐*O*‐PEG_3_‐BBN_7‐14_).

**3** was completely built on solid support using standard Fmoc‐based solid phase‐assisted synthesis protocols (Scheme 1 B). **2** was also in part synthesized by solid phase‐assisted protocols (up to the *bis*‐amine PEG_1_‐*d*‐Cys(S‐S‐*t*Bu)‐TrL, **1**), but also further modified in solution to obtain **2. 1** was synthesized on a rink amide resin using a short PEG linker to increase product yields and purities of the dendritic system, *t*Bu‐thio‐protected *d*‐ cysteine to increase the resistance of the final molecule towards metabolic degradation and the symmetrically branching building block *N*,*N*‐bis(*N*′‐Fmoc‐3‐aminopropyl)‐glycine potassium hemisulfate ((Fmoc‐NH‐Propyl)_2_‐Gly‐OH) to introduce two fully equivalent amino functionalities for further modification. After cleavage from the resin with TFA/TIS (5 %), leaving the *t*Bu‐thio‐protecting group of the cysteine‐thiol intact, and purification, **1** was reacted in solution with succinimidyl‐*p*‐formyl‐benzoate (SFB, **a**) to give *bis*‐aldehyde **2** (Scheme 1 A).

By reacting, in an one‐pot synthesis step, first 2.25 equiv. of aminooxy‐PESIN **3** with *bis*‐aldehyde **2** (yielding intermediate PEG_1_‐*d*‐Cys(S‐S‐*t*Bu)‐TrL(PEG_3_‐BBN_7‐14_)_2_), followed by deprotection of the cysteine thiol with *tris*(2‐carboxyethyl)‐phosphine hydrochloride (TCEP), the thiol‐modified dimeric PESIN backbone **4** (PEG_1_‐*d*‐Cys(SH)‐TrL(PEG_3_‐BBN_7‐14_)_2_ was obtained. The intermediate PEG_1_‐*d*‐Cys(S‐S‐*t*Bu)‐TrL(PEG_3_‐BBN_7‐14_)_2_ could also be isolated but the one‐pot reaction to form **4** turned out to be more efficient. One major impurity which was always detected was the side product containing only one PESIN peptide instead of two, indicating that the synthesis of heterobivalent peptide shuttles would also be easily possible using this synthesis strategy. In the next step, **4** was further modified by a thiol‐based Michael click reaction with **b** (1,2‐*bis*‐maleimidoethane (BME))^[22]^ to obtain the final maleimide‐functionalized homodimeric peptide targeting vector **5** ((PEG_1_‐*d*‐Cys(S(BME))‐TrL(SFB‐PEG_3_‐BBN_7‐14_)_2_).

**Figure 1:**
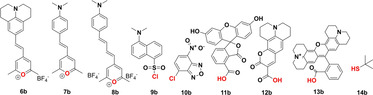
Depiction of the dyes **6 b**–**13 b** used to synthesize the MIUs **6 a**–**13 a** and the final multimodal imaging agents **6**–**13**. As a reference compound for the multimodal imaging agents, **14** (lacking both the dye and the chelator NODA‐GA) was synthesized from 2‐methyl‐2‐propan‐thiol **14 b**. The functional group of the respective dye which was reacted with the *N*_*ϵ*_‐amine of lysine to obtain the MIUs is marked red.

**Scheme 2:**
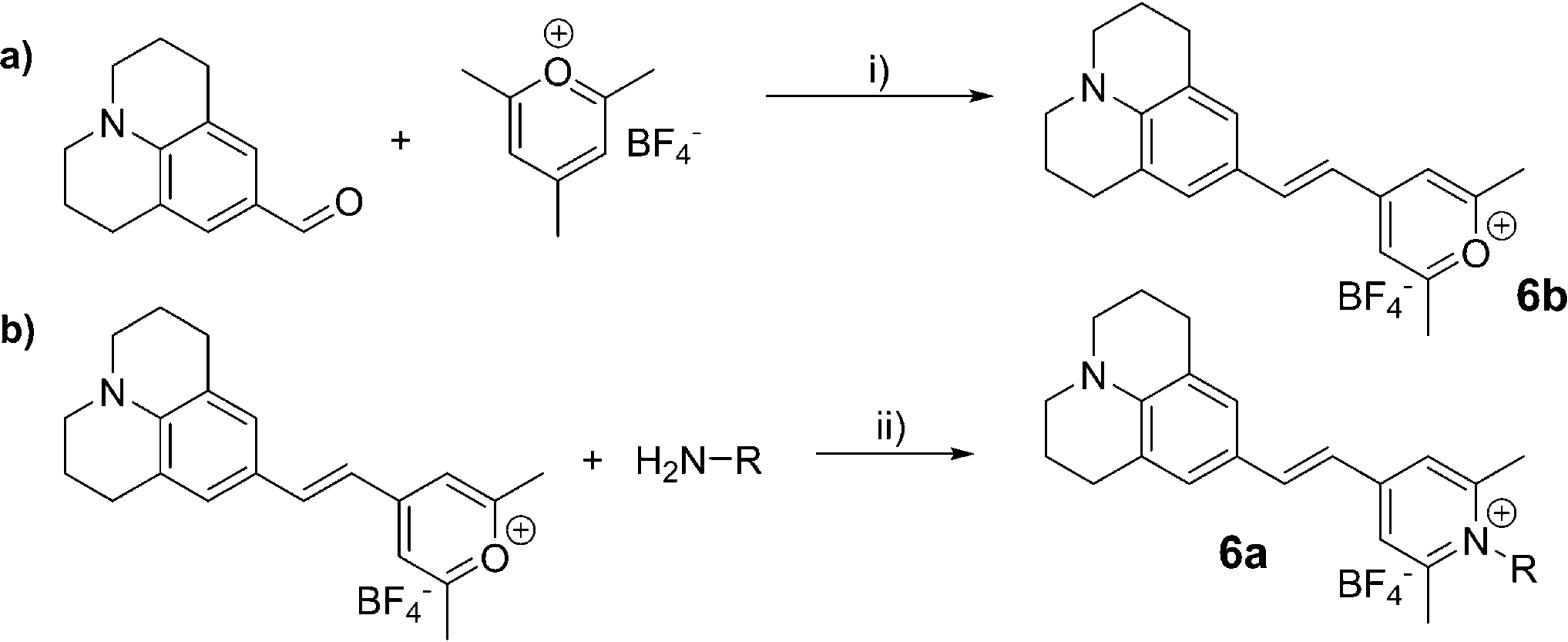
Schematic representation of a) the synthesis of the pyrilium‐dye **6 b**. Reaction conditions: i) MeOH, 65 °C for 10 min; evaporation of the solvent, purification by column chromatography on silica (CH_3_Cl/MeOH 4:1). b) conjugation of the pyrilium dye to the *N*_*ϵ*_‐amine of the lysine to form the pyridinium‐conjugate **6 a**.

The synthesis of the MIUs **6 a**–**13 a**, consisting of the NODA‐GA chelator, the fluorescent dye and a thiol group for PESIN‐dimer conjugation could be completely performed on solid support (Scheme 1 C). For this purpose, an orthogonal protecting group strategy had to be employed. First, the NODA‐GA‐modified di‐peptide Cys(Trt)‐Lys(alloc)‐NODA‐GA(*t*Bu)_3_ was built, followed by deprotection of the *N*_*ϵ*_‐alloc protecting group of lysine by palladium catalysis. Afterwards, the dyes **6 b**–**13 b** (Figure 1) were reacted with the free *N*_*ϵ*_‐amino functionality of the lysine. For this, the pyrilium dyes[^18h^, ^23^] **6 b**–**8 b** and the chlorides **9 b** and **10 b** were reacted directly (Scheme 2), dyes **11 b**–**13 b** were activated before using HBTU.[^10d^, ^18g^] After cleavage from solid support and deprotection, the MIUs **6 a**–**13 a** were obtained.

As mentioned before, the convergent synthesis strategy to synthesize an appropriately functionalized biomolecule (in our case, the maleimide‐modified PESIN‐dimer **5**) as well as the functionalized MIUs (**6 a**–**13 a**) apart from each other enables on the one hand the efficient formation of the complex final multimodal imaging agents **6**–**13** which is not possible by following a divergent synthesis strategy. In addition, this modular strategy, based on the “molecular Lego concept”,^[24]^ exhibits some further advantages. In our study, each MIU could be investigated regarding its chemical, radiochemical and photophysical properties, enabling to determine the influence of the individual dye on the mentioned properties of the respective MIU **6 a**–**13 a**. The same holds true for the respective bioconjugates **6**–**13**. Only by this, we were able to identify structure–activity‐relationships (SARs) for the different multimodal imaging agents and to determine the influence of each molecular building block on the global properties of the compounds. For future applications, this modular approach furthermore offers an easy way to conjugate the described MIUs to other peptidic shuttles (monomeric, homodimeric or even heterbi‐ or multivalent) or antibodies to address other target receptors. This offers the possibility to develop individualized imaging agents adapted to the respective tumor entity.

To assemble the multimodal imaging agents **6**–**13**, the thiol groups of the MIUs **6 a**–**13 a** were reacted with the maleimide of the peptide dimer **5** in a click chemistry Michael addition reaction, resulting in the formation of the bioconjugates within only 5 minutes.^[22b]^ As we intended to shed light on the influence of the MIUs on the chemical, biological and photophysical properties of the resulting bioconjugates, the synthesis of appropriate reference compounds was mandatory. Thus, we further synthesized **14** from **5** and 2‐methyl‐2‐propanethiol (**14 b**), lacking both chelator and dye to investigate the influence of these structure elements on the mentioned properties of the bioconjugates. In addition, we synthesized other reference compounds, comprising only a small methyl‐thiol instead of the MIUs (**CC5**) and a conjugate bearing only the NODA‐GA chelator but lacking the fluorescent dye (**CC4**) and furthermore also designed bioconjugates exhibiting the same structural design as **6**–**13** but comprising near‐infrared fluorescent indocyanine (ICG) dyes (**CC1**–**CC3**) (Scheme S1) (detailed data for **CC1**–**CC5**, comprising syntheses and full characterization, are described elsewhere^[25]^ but some results obtained for these compounds were included here to round off the SAR analysis and to complete the picture).

**Figure 2:**
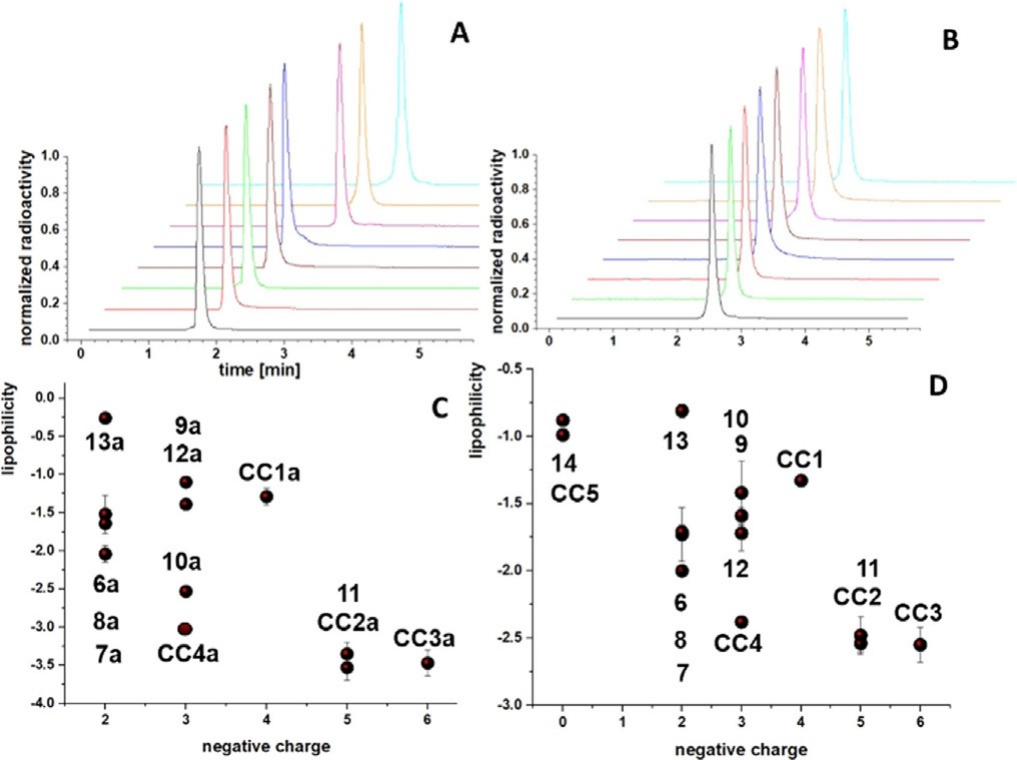
**A**: Radio‐HPLC chromatograms of the MIUs ^68^Ga‐**6 a**–^68^Ga‐**13 a** (front to back: ^68^Ga‐**9 a**, ^68^Ga‐**10 a**, ^68^Ga‐**11 a**, ^68^Ga‐**7 a**, ^68^Ga‐**8 a**, ^68^Ga‐**12 a**, ^68^Ga‐**6 a** and ^68^Ga‐**13 a**). **B**: Radio‐HPLC chromatograms of the multimodal imaging agents ^68^Ga‐**6**–^68^Ga‐**13** (front to back: ^68^Ga‐**9**, ^68^Ga‐**11**, ^68^Ga‐**10**, ^68^Ga‐**8**, ^68^Ga‐**7**, ^68^Ga‐**12**, ^68^Ga‐**6** and ^68^Ga‐**13**). **C**: Plot of lipophilicity vs. overall charge of the MIUs **6 a**–**13 a** and **CC1 a**–**CC4 a. D**: Plot of lipophilicity vs. overall charge of the bioconjugates **6**–**14** and **CC1**–**CC5**.

**Scheme 3:**
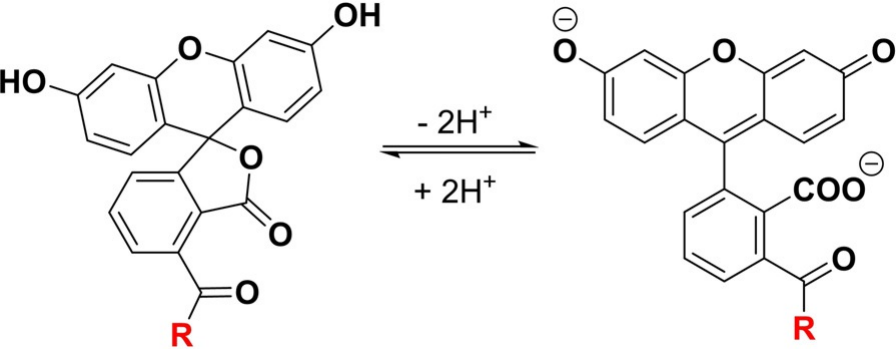
Lactone ring opening of fluorescein and resonance‐stabilized quinone structure.

Following the synthesis of the MIUs **6 a**–**13 a** and the corresponding bioconjugates **6**–**13**, the radiolabeling efficiency of the compounds with ^68^Ga^3+^ was investigated. Radiolabeling of all compounds under investigation was performed under standard conditions (pH 4; 45 °C, 10 min) and the corresponding ^68^Ga‐labeled products were obtained in high radiochemical yields (RCYs) and radiochemical purities (RCPs) of ≥95 % and in high non‐optimized molar activities of 90–120 GBq μmol^−1^ (Figure 2 A). As the MIUs **6 a**–**13 a** contain unprotected thiol functionalities, it demonstrated to be mandatory to add the reductive agent TCEP to the radiolabeling mixtures to prevent ‐S‐S‐ dithiol‐formation. The dimerization reaction could be fully suppressed and also completely reversed using TCEP. As expected, no addition of TCEP was necessary to obtain homogeneous [^68^Ga]**6**–[^68^Ga]**13** as their precursors do not contain free thiols which could result in dimer formation upon radiolabeling. [^68^Ga]**6**–[^68^Ga]**13** could be obtained under the same mild radiolabeling conditions and in the same high RCPs, RCYs and molar activities as their corresponding MIUs (Figure 2 B) and showed no fragmentation or otherwise degradation over time, indicating a high chemical stability of the compounds. In the following, we investigated the lipophilicity of the MIUs [^68^Ga]**6 a**–[^68^Ga]**13 a** and their bioconjugates [^68^Ga]**6**–[^68^Ga]**13** (Table 1 and Figure 2 C,D). The log_*D*_ data obtained showed a strong dependence of the compound's hydrophilicity/ lipophilicity on the structural composition of the molecules and especially on their charge (Figure 2 C,D). The exceptionally low log_*D*_ of **11 a** and **11** can be attributed to the opening of the lactone ring of the fluorescein molecule, resulting in resonance stabilization in the quinone form (Scheme 3) and the by this significantly increased charge of the system. From the log_*D*_ data, it can be deduced that the MIUs **6 a**–**13 a** decide the magnitude of the lipophilicity of the whole bioconjugates **6**–**13**. Taking further the data of reference compounds (**CC4 a**=−3.35±0.15, **CC4**=−2.38±0.03, **CC5**=−0.99±0.03 and **14** (Table 1)) into account, it becomes clear that the main influence on the lipophilicity of the whole bioconjugate constructs is exerted by the applied fluorescent dye (**6 b**–**13 b**) and not by the peptides or the chelator. This is important as the lipophilicity significantly influences the in vivo elimination pathway of the respective substance.^[26]^ This offers a possibility to precisely tailor the characteristics of hybrid multimodal imaging agents by choosing the appropriate fluorescent dye.

**Table 1:** Summary of the log_*D*_ data of the MIUs **6 a**–**13 a** and their corresponding bioconjugates **6**–**14** as well as the GRPR affinity data (IC_50_ values) of **6**–**14**.

Compound	log_*D*_	Overall negative charge	IC_50_ [nm]
**6 a**	−1.64±0.05	2	
**6**	−1.71±0.05	2	18.99±1.13
**7 a**	−2.04±0.11	2	
**7**	−2.00±0.01	2	16.47±0.25
**8 a**	−1.54±0.25	2	
**8**	−1.73±0.20	2	15.51±0.76
**9 a**	−1.39±0.04	3	
**9**	−1.59±0.06	3	26.20±0.89
**10 a**	−2.53±0.07	3	
**10**	−1.42±0.24	3	25.19±2.82
**11 a**	−3.43±0.16	5	
**11**	−2.54±0.06	5	62.07±3.87
**12 a**	−1.10±0.07	3	
**12**	−1.72±0.13	3	20.91±1.67
**13 a**	−0.26±0.05	2	
**13**	−0.81±0.05	2	17.96±0.86
**14**	−0.88±0.04	0	18.67±1.37

**Figure 3:**
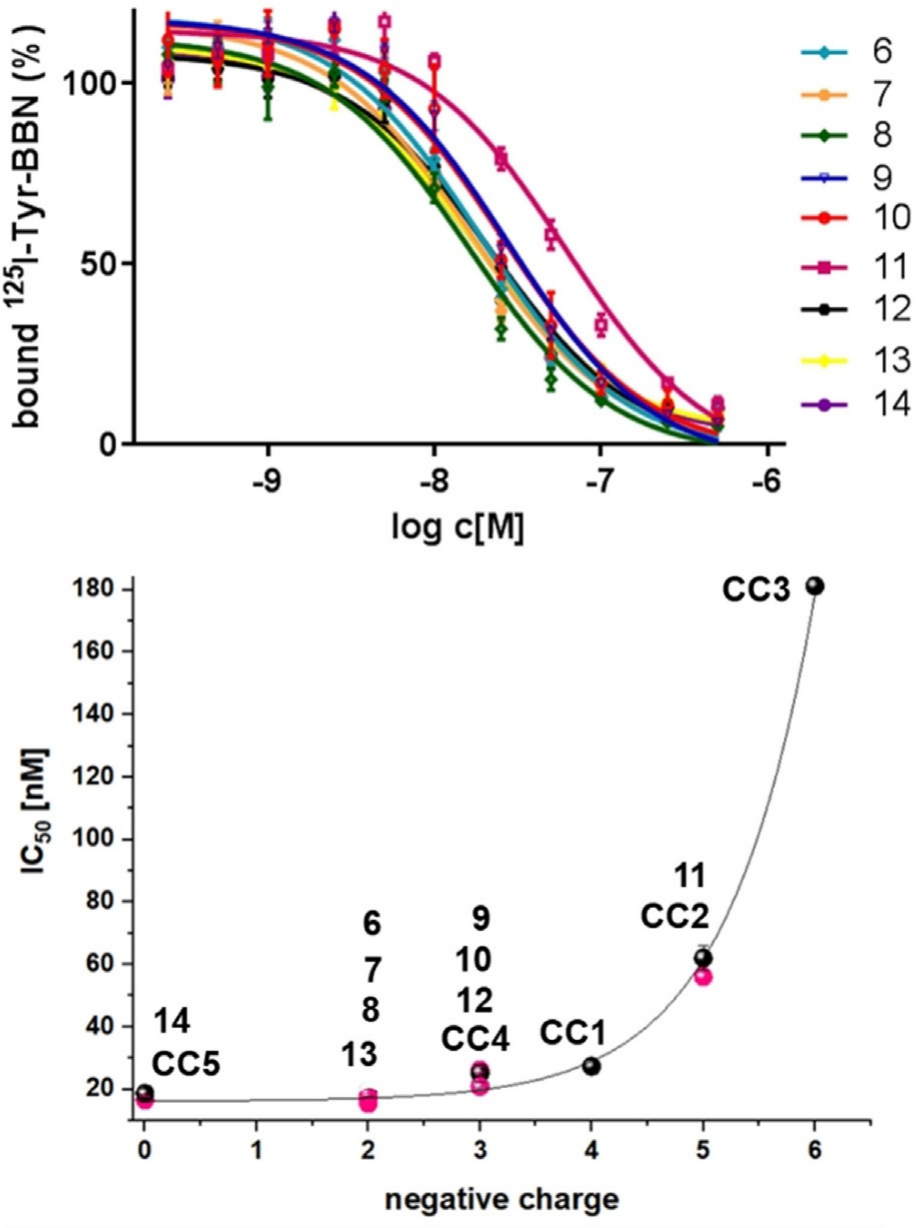
Results of the competitive displacement assays of the bioconjugates **6**–**14** on stably GRPR‐transfected HEK cells using [^125^I]‐Tyr^4^‐bombesin as competitor. Upper panel: Binding curves for **6**–**14**. Lower panel: Correlation between IC_50_ values and number of overall negative charges of **6**–**14** (red) and **CC1**–**CC5** (black).

To further determine the influence of the MIUs not only on the chemical, but also on the biological properties of the resulting bioconjugates, the GRP receptor affinities of **6**–**14** were investigated in a competitive displacement assay on stably GRPR‐transfected HEK cells (HEK‐GRPR) using [^125^I]‐Tyr^4^‐bombesin as competitor. All bioconjugates showed IC_50_ affinity data in the range of 15.51±0.76 nm to 62.07±3.87 nm (Table 1 and Figure 3).

Endogenous bombesin (BBN) was used as internal reference and showed an IC_50_ value of 2.81±0.56 nm. From these data, several interesting trends can be identified: i) All bioconjugates showed a decreased GRPR binding affinity compared to the endogenous reference BBN, ii) Interestingly, the bioconjugates **6**, **7**, **8** and **13** (containing pyridinium dyes and rhodamine 101) demonstrated GRPR‐affinities (18.66±1.13 nm, 16.47±0.25 nm, 15.51±0.76 nm and 17.96±0.86 nm, respectively) being very similar to that of the reference **14** (18.67±1.37 nm), lacking both chelator and fluorescent dye. In these bioconjugates, the applied fluorescent dyes exhibit a positive charge. iii) Correspondingly, the group of bioconjugates carrying an overall uncharged fluorescent dye (**9**, **10** and **12**) also showed similar, slightly decreased GRPR affinity values of 26.20±0.89 nm, 25.19±2.82 nm and 20.91±1.67 nm, respectively, iv) The only bioconjugate carrying a twice negatively charged fluorescein dye (**11**) shows a considerably decreased GRPR affinity (IC_50_ value of 62.07±3.87 nm).

Taken together, the charge of the fluorescent dye introduced into the MIUs and in the following also into the corresponding bioconjugates seems to be the factor mainly influencing the biological receptor binding properties of the resulting multimodal imaging agents. The reference compound **14**, lacking both chelator and dye showed a GRPR‐affinity comparable to the multimodal imaging agents **6**, **7**, **8** and **13** comprising positively charged dyes, thus indicating that neither the introduction of the chelator or fluorescent dye per se result in a loss of receptor affinity but seem to have—if at all—only a minor influence on the resulting receptor binding characteristics. In contrast, with increasing number of negative charges in the dye molecule, the binding affinity considerably decreases, indicating a much higher influence of charge than of molecular size and composition of the MIUs on the receptor affinities of the corresponding bioconjugates.

This conclusion is supported by the GRPR affinity data that were obtained for the ICG‐comprising bioconjugates **CC1**–**CC3** and the reference compounds **CC4** and **CC5** ^[25]^: Also within the group of ICG‐comprising bioconjugates **CC1**–**CC3**, a strong correlation between the number of negative charges of the respective dye molecule and GRPR affinity decrease was observed, going from **CC1** (27.39±2.01 nm; one negative charge on the ICG dye) over **CC2** (56.07±1.47 nm; two negative charges) to **CC3** (181.23±2.24 nm; three negative charges). The binding data obtained for the reference compounds **CC4** (carrying only a NODA‐GA chelator and no dye; IC_50_ of 21.48±1.20 nm) and **CC5** (carrying neither chelator nor dye; IC_50_ of 16.64±1.06 nm) support the conclusions drawn before.

This indicates a clear influence of the sum net charge of the introduced fluorescent dye on the GRPR binding capability and no or only an insignificant impact of the spatial demand of the chelator, dye or dye‐chelator‐complex. The choice of a suitably charged dye thus offers another possibility to tailor the characteristics of hybrid multimodal imaging agents regarding target receptor binding and thus in vivo pharmacokinetics.

After studying the chemical and biological properties of the MIUs and their corresponding multimodal imaging agents, the photophysical properties of the substances were finally under investigation. Here, we found no significant decrease or shift of the optical and fluorescent characteristics of the bioconjugates compared to the MIUs (Table 2).

**Table 2:** Summary of the photophysical properties of the MIUs **6 a**–**13 a** and the hybrid bioconjugates **6**–**13**. (values were determined in PBS at a concentration of 1×10^−5^ mol L^−1^).

Compound	*λ*_max_ [nm]	Log *ϵ* [m ^−1^ cm^−1^]	λ_em_^[a]^ [nm]	Stokes shift [nm]
**6 a**	480	4.17	635	155
**6**	510	4.08	630	130
**7 a**	440	4.16	600	160
**7**	460	4.23	600	140
**8 a**	440	4.26	700	260
**8**	470	4.16	700	230
**9 a**	–	–	550	–
**9**	–	–	540	–
**10 a**	480	4.31	550	70
**10**	490	4.10	545	60
**11 a**	450	4.18	520	70
**11**	450	4.02	525	65
**12 a**	445	4.25	495	50
**12**	455	4.30	495	40
**13 a** ^[b]^	585	4.50	605	20
**13 b**	590	4.57	610	20

[a] excitation wavelength λ_ex_=400 nm. [b] excitation wavelength λ_ex_=500 nm

**Figure 4:**
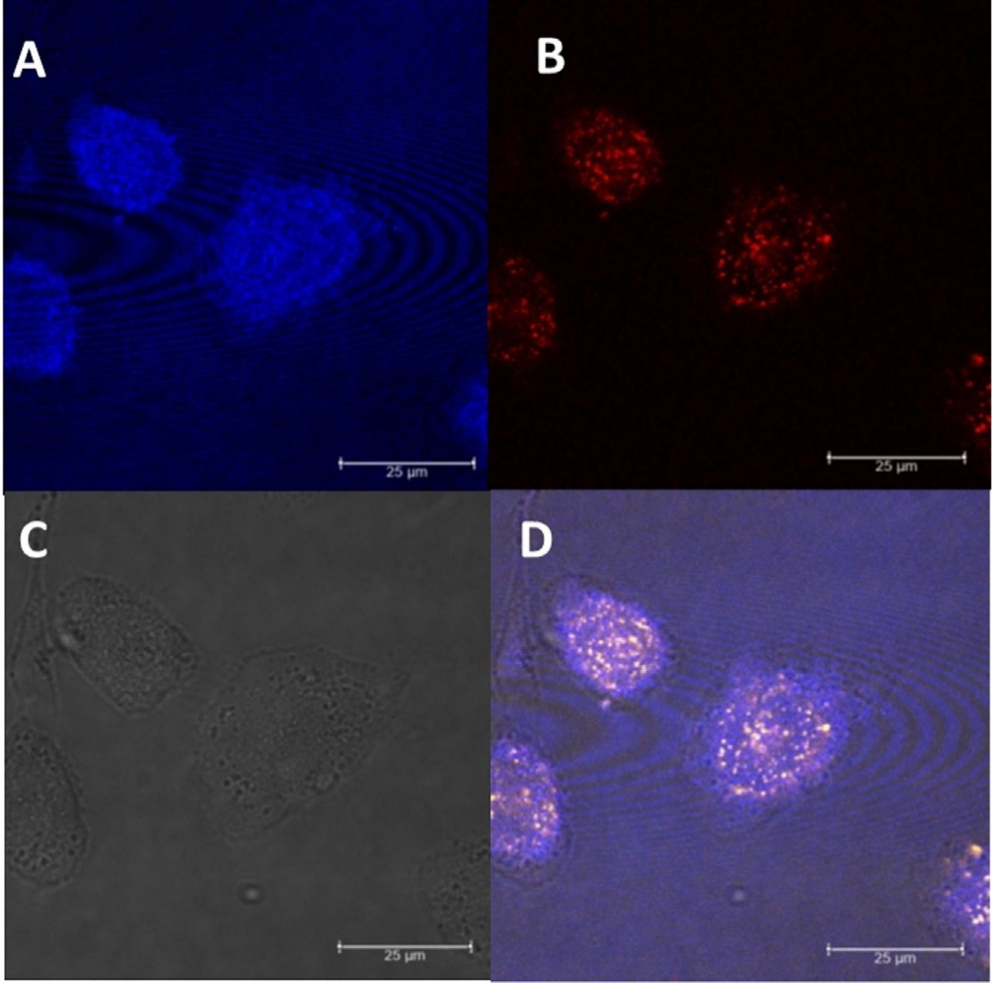
Confocal fluorescence microscopy images for pyridinium‐based bioconjugate **6** (**A–D**). (**A**) Detector window (440–490 nm, DAPI). (**B**) NIR detector window (660–720 nm, **6**). (**C**) Transmitted light. (**D**) Merged image of all 3 pictures.

Rather exceptional but favorable fluorescence characteristics were found for the pyridinium‐based bioconjugates **6**, **7** and **8** as they were shown to exhibit large Stokes Shifts of 130 nm for **6**, 140 nm for **7** and even 230 nm for **8**, respectively.[^18h^, ^23^] For **6**, initial confocal microscopy experiments were performed on GRPR‐positive PC‐3 cancer cells and revealed—as expected—a cytoplasmic localization of the agent (Figure 4) and a stable fluorescence emission even after prolonged exposure (detector window 660–720 nm).^[18d]^

## Conclusions

We aimed here to design multimodal imaging units being composed of a chelator for PET radiometal nuclide labeling, a fluorescent dye and a chemoselectively and efficiently reacting functional group. These were introduced into a symmetrically branched PESIN homodimer serving as target‐specific vector, resulting in GRPR‐specific multimodal imaging agents. In the following, we determined how the different fluorescent dyes introduced influence chemical, biological and photophysical parameters of the final bioconjugates and found that: i) the lipophilicity of the MIUs as well as their bioconjugates strongly depends on the solubility properties of the respective fluorescent dye and only to a minor extent on the other molecular building blocks (chelator and peptide dimer), ii) the radiolabeling properties with the β^+^‐emitter ^68^Ga^3+^ were not found to be influenced by the introduced dyes although different sets of heteroatom donors are present. Labeling of all conjugates could be achieved in high RCYs, RCPs and molar activities, iii) the GRPR binding capability of the bioconjugates is not influenced by the introduction of the NODA‐GA chelator or a fluorescent dye per se, but strongly depends on the sum of the net charge of the applied dye. We found a strong correlation between the number of negative charges and the decrease in GRP receptor affinity. iv) no significant decrease or alteration of the photophysical properties for neither the MIUs nor the bioconjugates was observed.

Taken together, neither the ^68^Ga‐radiolabeling nor the photophysical properties of the described complex systems are influenced by any of the molecular units forming together the multimodal imaging agents. In contrast, log_*D*_ values and GRP receptor binding characteristics strongly depend on the choice of the respective fluorescent dye which gives a perfect tool in hand to tailor the properties of hybrid multimodal imaging agents on the basis of PESIN homodimers regarding their intended application for multimodal PET/OI imaging of malignant tissue.

## Experimental Section

Experimental details, synthetic procedures, analytical data and photophysical investigations are provided in the supporting information.

## Conflict of interest

The authors declare no conflict of interest.

## Supporting information

As a service to our authors and readers, this journal provides supporting information supplied by the authors. Such materials are peer reviewed and may be re‐organized for online delivery, but are not copy‐edited or typeset. Technical support issues arising from supporting information (other than missing files) should be addressed to the authors.

Supplementary
