# Supplementary material for: Functional Hybrid Molecules for the Visualization of Cancer: PESIN‐Homodimers Combined with Multimodal Molecular Imaging Probes for Positron Emission Tomography and Optical Imaging: Suited for Tracking of GRPR‐Positive Malignant Tissue
Source: Chemistry. 2020 Oct 28;26(69):16349–56. doi: 10.1002/chem.202002386 (PMC7756681; doi:10.1002/chem.202002386)
Supplement: Supplementary file 1 — Supplementary [file CHEM-26-16349-s001.pdf]

Chemistry–A European Journal

Supporting Information

Functional Hybrid Molecules for the Visualization of Cancer: PESIN-Homodimers Combined with Multimodal Molecular Imaging Probes for Positron Emission Tomography and Optical Imaging: Suited for Tracking of GRPR-Positive Malignant Tissue**

Ralph Hübner,*[a] Xia Cheng,[b] Björn Wängler,[b] and Carmen Wängler*[a]

Content

Scheme 1 Depiction of the structures and syntheses of **CC1 – CC5** page 2

Experimental Section:

General, syntheses and analytical data of **1 – 14**, **6a – 13a** and **6b – 8b** page 3

Radiochemistry page 22

Log_D determination page 22

Competitive receptor binding assay page 22

Confocal Fluorescence Microscopy page 23

References page 23

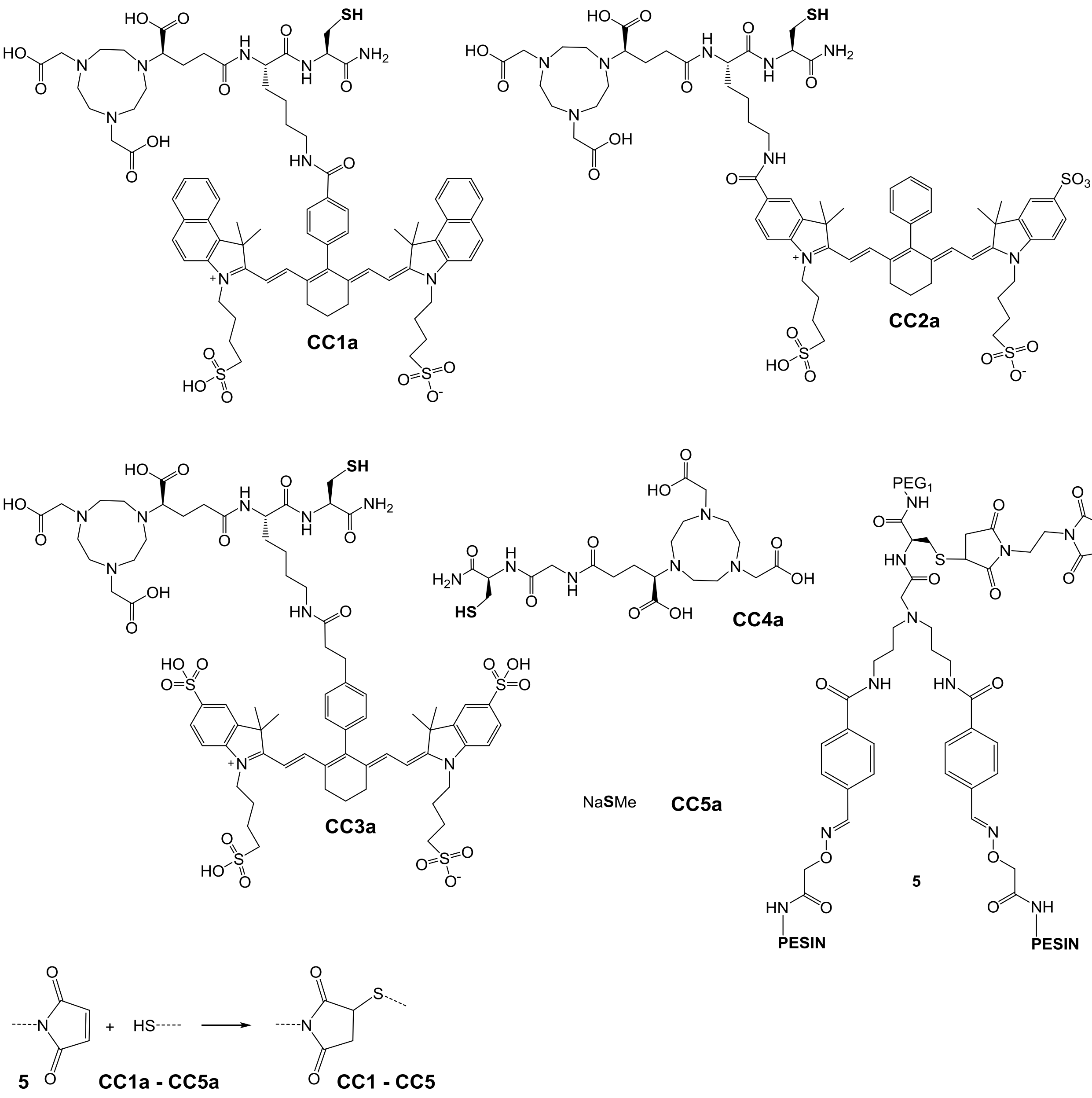

Scheme S1 Depiction of the structures and syntheses of **CC1 – CC5**

Experimental Section

General

All commercially available chemicals and solvents were at least of analytical grade and used, if not otherwise stated, without further purification. Fmoc-protected amino acids and rink amide resin (loading = 0.52 mmol/g) were purchased from NovaBiochem. 15-(9-Fluorenylmethyloxy-carbonyl)amino-4,7,10,13-tetraoxa-pentadecanoic acid (PEG_3, Fmoc-NH-PEG_3-COOH), 8-(9-fluorenylmethyloxycarbonyl-amino)-3,6-dioxaoctanoic acid (PEG_1, Fmoc-NH-PEG_1-COOH), *N*-alpha-(9-fluorenylmethyloxycarbonyl)-S-(*t*Bu-thio)-*D*-cysteine (Fmoc-*D*-Cys(S-S-*t*Bu)-OH), {[*bis*(*t*-butyloxycarbonyl)amino]oxy}acetic acid monohydrate ($(Boc)_2$AOAc-OH x H_2O) and 2-(*bis*(3-(((9*H*-fluoren-9-yl)methoxy)carbonylamino)propyl)amino)acetic acid potassium hemisulfate (TrL, Fmoc-NH-$Propyl)_2$Gly-OH x $KHSO_4$) were obtained from Iris Biotech, *N*-succinimidyl-4-formylbenzoate (95 %) (SFB) from ABCR and *tris*(2-carboxyethyl)phosphine hydrochloride (TCEP) from Alfa Aesar. Dichloromethane (DCM), diethylether, dimethylformamide (DMF), 2-(1*H*-benzotriazol-1-yl)-1,1,3,3-tetramethyluronium hexafluoro-phosphate (HBTU), trifluoroacetic acid (TFA) and water were purchased from Carl Roth, acetonitrile (MeCN) from Häberle Labortechnik, *N,N*-diisopropylethylamine (DIPEA), triisopropylsilane (TIS) and 5-(dimethylamino)naphthalene-1-sulfonyl chloride (Dansyl-Chloride), 4-chloro-7-nitro-1,2,3-benzoxadiazole (NBD-Chloride), 9-(2-carboxyphenyl)-2,3,6,7,12,13,16,17-octahydro-1*H*,5*H*,11*H*,15*H*-xantheno[2,3,4-ij:5,6,7-i'j']diquinolizin-18-ium, inner salt (Rhodamin 101 inner salt), 2,3,6,7-tetrahydro-11-oxo-1*H*,5*H*,11*H*-[1]benzopyrano[6,7,8-ij]quinolizine-10-carboxylic acid (Coumarin 343), 5(6)-carboxyfluorescein *N*-hydroxysuccinimide ester and 2-methyl-2-propan-thiol from Sigma-Aldrich, 1,2-*bis*(maleimido)ethane (BME), 2,4,6-trimethylpyrylium tetrafluoroborate, 4-dimethylaminobenzaldehyde, 4-dimethylaminocinnamaldehyde, 9-julolidinecarboxaldehyde and *tetrakis*(triphenylphosphine)palladium(0) ($Pd(PPh_3)_4$) from TCI and 4-(4,7-*bis*(2-(*t*-butoxy)-2-oxoethyl)-1,4,7-triazacyclononan-1-yl)-5-(tert-butoxy)-5-oxopentanoic acid ((*R*)-NODA-GA(*t*$Bu)_3$) from CheMatech.

For HPLC chromatography, a Dionex UltiMate 3000 system was used together with Chromeleon Software (Version 6.80). For analytical chromatography, a Chromolith Performance (RP-18e, 100-4.6 mm, Merck, Germany) and for semipreparative analyses, Chromolith (RP-18e, 100-10 mm, Merck, Germany) columns were used, respectively. For radioanalytical use, a Dionex UltiMate 3000 system equipped with a Raytest GABI Star radioactivity detector was used together with a Chromolith Performance (RP-18e, 100–4.6 mm, Merck, Germany) column. All operations were performed with a flow rate of 4 mL/min using H_2O + 0.1% TFA and MeCN + 0.1% TFA as solvents. HR-ESI (high-resolution Electrospray Ionization) and MALDI (Matrix-Assisted Laser Desorption/Ionization) spectra were obtained with Finnigan MAT95Q and Bruker Daltronics Microflex spectrometers, respectively. γ-counting was performed using a 2480 Wizard gamma counter system from Perkin Elmer. A Cary 100 Bio system (Varian) was used to record the UV/Vis-Spectra together with 4 mL PMMA cuvettes from Sigma-Aldrich. Further, for absorbance and emission measurements, a Tecan Infinite M200 Microplate reader together with a Nunc Micro-Well 96 solid plate from ThermoFisher was used. Confocal fluorescence

microscopy was performed on a Leica TCS SP8 confocal microscope with excitation lasers at λs of 488, 552 and 638 nm. Overlays of microscopies were generated directly with the operating software or afterwards using FIJI software (V1.50e).

Transfected Human Embryonic Kidney 293 cells stably expressing the GRP-Receptor (HEK-GRPR) were used for cells experiments. HEK-GRPR cells were obtained from Dr. Martin Béhé, Paul Scherrer Institute, Villingen, Switzerland. The human tumor cell line PC-3 (GRPR-positive) was obtained from DSMZ. [^{125}I]-Tyr^{4}-bombesin was purchased from Perkin Elmer (NEX258010UC, molar activity: 81.4 GBq/µmol). Dulbecco's Modified Eagle's Medium (DMEM, high glucose, GlutaMax-I, 500 mL), Geneticin (G418 Sulfate, 50 mg/mL), Opti-MEM I (GlutaMAX I), RPMI 1640 medium, L-Glutamine and PenStrep were obtained from Gibco, FCS (fetal calf serum) from BioCell and Dulbecco's phosphate buffered saline (PBS), 0.25% Trypsin and 0.02% EDTA Solution in PBS from Sigma-Aldrich. The ^{68}Ge/^{68}Ga-Generator used was an IGG100 system, obtained from Eckert & Ziegler, Berlin, Germany and eluted with HCl (0.1 M, 1.6 mL).

Syntheses

General: The PESIN peptide sequence (H_2N-O-(CH_2)-(CO)-HN-PEG_3-Gln-Trp-Ala-Val-Gly-His-Leu-Met-NH_2) **3**, Cys(Trt)-Lys(alloc)-NODA-GA(*t*Bu)$_3$, PEG_1-*D*-Cys(S-S-*t*Bu)-TrL-(Fmoc)$_2$ and PEG_1-*D*-Cys(S-S-*t*Bu)-TrL-(NH_2)$_2$ (**1**) were synthesized by standard Fmoc-based solid phase peptide synthesis protocols[1] and subsequent conjugation of the respective amino acids. Cleaving from resin was usually done with a mixture of 95 % TFA and 5 % TIS for 1 – 2 h by ambient temperature followed by evaporation of the volatile materials, the residue was dissolved in 1:1 MeCN:H_2O + 0.1 % TFA and subsequently purified by semipreparative HPLC. The syntheses of the pyrylium dye molecules **6b** – **8b** was carried out according to the reported procedure.[2]

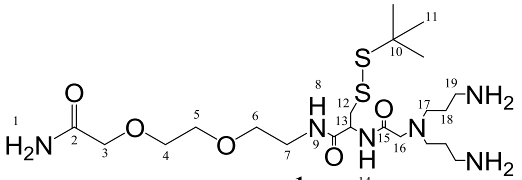

1

1: After deprotection, cleavage from resin and purification via HPLC PEG_1-*D*-Cys(S-S-*t*Bu)-TrL-(NH_2)$_2$ (**1**) was obtained as a colorless solid material. ($C_{21}H_{44}N_6O_5S_2$): HPLC gradient (semipreparative): 0 – 100 % MeCN + 0.1 % TFA in 5 min, R_t = 3.00 min. HPLC gradient (analytical): 0 – 100 % MeCN + 0.1 % TFA in 5 min, R_t = 1.12 min, yield: 75 %, purity: 95 %, MALDI-MS (m/z) for $[M+H]^+$ (calculated): 524.45 (524.28); $[M+Na]^+$ (calculated): 546.47 (546.26); $[M+K]^+$ (calculated): 562.36 (562.24); HR-ESI-MS (*m/z*) for $[M+H]^+$ (calculated): 525.2885 (525.2893). ^{1}H-NMR (400 MHz, Acetonitrile-d$_3$): δ = 8.20 (d, ^3J = 6.9 Hz, 1H, 14-H), 7.41 (t, ^3J = 5.4 Hz, 1H, 8-H), 7.15 (s, 1H, 1-H), 6.76 (s, 1H, 1`-H), 4.61 (ddd, ^3J = 8.4, 6.9, 4.9 Hz, 1H, 13-H), 3.98 (s, 2H, 3-H), 3.76 – 3.68 (m, 2H, 6-H), 3.69 – 3.66 (m, 2H, 5-H), 3.62 – 3.60 (m, 2H, 4-H), 3.57 – 3.54 (m, 2H, 7-H), 3.48 – 3.28 (m, 2H, 16-H), 3.17 (dd, ^2J = 13.6, ^3J = 4.9 Hz, 1H, 12-H), 3.07 (m, 8H, 17/19-H), 2.99 (dd, ^2J = 13.6, ^3J = 8.4 Hz, 1H, 12`-H), 2.06 (m, 4H,

18-H), 1.35 (s, 9H, 11-H). ^{13}C NMR (101 MHz, CD_3CN) δ = 174.79 (1C, C_q, C-2), 170.47 (1C, C_q, C-9), 161.23 (1C, C_q, C-15), 71.28 (1C, CH_2, C-3), 70.39 (2C, CH_2, C-4), 70.26 (2C, CH_2, C-5), 69.62 (1C, CH_2, C-6), 55.66 (1C, C_q, C-10), 53.94 (1C, CH, C-13), 52.95 (2C, CH_2, C-17), 48.41 (1C, CH_2, C-16), 42.52 (1C, CH_2, C-7), 39.89 (1C, CH_2, C-12), 38.49 (2C, CH_2, C-19), 29.64 (3C, CH_3, C-11), 23.19 (2C, CH_2, C-18).

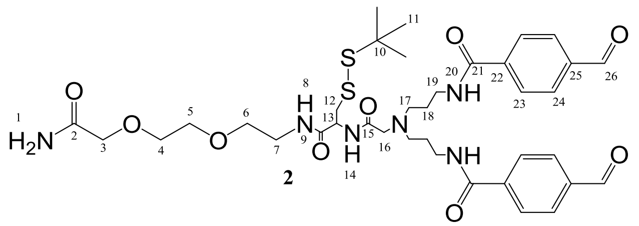

2

2: 1 eq. **1** (1 mg) was dissolved in 1:1 MeCN:H_2O + 0.1 % TFA and 2 eq. of SFB (0.94 mg), dissolved in MeCN + 0.1 % TFA, were added. The pH of the mixture was adjusted to 7.0 by adding phosphate buffer solution (0.5 M, pH = 7.2). The reaction mixture was stirred at 50°C for 30 minutes and afterwards, the reaction vessel was directly frozen with liquid nitrogen and freeze-dried overnight. The residue was dissolved in 1:1 MeCN:H_2O + 0.1 % TFA, where a phase separation took place. The organic layer was subsequently purified by HPLC to give **2** as a white solid. ($C_{37}H_{52}N_6O_9S_2$): HPLC gradient (semipreparative): 25 – 65 % MeCN + 0.1 % TFA in 8 min, R_t = 4.00 min; 0 – 100 % MeCN + 0.1 % TFA in 12 min, R_t = 6.40 min. HPLC gradient (analytical): 0 – 100 % MeCN + 0.1 % TFA in 5 min, R_t = 2.00 min, yield: 85 %, purity: 95 %, MALDI-MS (m/z) for $[M+H]^+$ (calculated): 788.91 (789.32); $[M+Na]^+$ (calculated): 810.90 (810.31); $[M+K]^+$ (calculated): 826.86 (826.28); HR-ESI-MS (*m/z*) for $[M+H]^+$ (calculated): 789.3310 (789.3237). ^1H-NMR (500 MHz, Acetonitrile-d_3): δ = 10.07 (s, 2H, 26-H), 8.30 (d, ^3J = 8.7 Hz, 1H, 14-H), 7.99 (d, ^3J = 8.2 Hz, 4H, 23-H), 7.94 (d, ^3J = 8.2 Hz, 4H, 24-H), 7.76 (s, 2H, 1-H), 7.20 (s, 1H, 20-H), 6.90 (s, 1H, 20`-H), 6.20 (s, 1H, 8-H), 4.70 (ddd, ^3J = 8.7, 8.4, 4.8 Hz, 1H, 13-H), 3.94 (s, 2H, 3-H), 3.69 – 3.65 (m, 2H, 6-H), 3.63 – 3.57 (m, 2H, 7-H), 3.52 (t, ^3J = 5.3 Hz, 4H, 4/5-H), 3.45 – 3.37 (m, 2H, 16-H), 3.30 (m, 4H, 17-H), 3.21 (dd, ^2J = 13.7, ^3J = 4.8 Hz, 1H, 12-H), 3.01 (dd, ^2J = 13.7, ^3J = 8.4 Hz, 1H, 12`-H), 2.40 (m, 4H, 19-H), 2.04 (m, 4H, 18-H), 1.34 (s, 9H, 11-H). ^{13}C NMR (101 MHz, CD_3CN) δ = 192.82 (2C, CH, C-26), 173.75 (1C, C_q, C-2), 169.91 (1C, C_q, C-9), 167.71 (2C, C_q, C-21), 160.64 (1C, C_q, C-15), 139.78 (2C, C_q, C-22), 138.93 (2C, C_q, C-25), 129.99 (4C, CH, C-23), 128.54 (4C, CH, C-24), 71.30 (1C, CH_2, C-3), 70.63 (2C, CH_2, C-4/5), 69.69 (1C, CH_2, C-6), 54.40 (1C, CH, C-13), 54.20 (1C, C_q, C-10), 53.85 (2C, CH_2, C-17), 48.35 (1C, CH_2, C-16), 42.74 (1C, CH_2, C-7), 39.78 (1C, CH_2, C-12), 36.86 (2C, CH_2, C-19), 29.66 (3C, CH_3, C-11), 24.99 (2C, CH_2, C-18).

4: ($C_{155}H_{226}N_{38}O_{43}S_3$): HPLC gradient (semipreparative): 25 – 65 % MeCN + 0.1 % TFA in 8 min, R_t = 3.70 min; 0 – 100 % MeCN + 0.1 % TFA in 12 min, R_t = 6.40 min. HPLC gradient (analytical): 0 – 100 % MeCN + 0.1 % TFA in 8 min, R_t = 3.30 min, yield: 75 %, purity: 95 %, MALDI-MS (m/z) for $[M+H]^+$ (calculated): 3405.13 (3404.59); $[M+Na]^+$ (calculated): 3426.96 (3426.57); $[M+K]^+$ (calculated): 3442.85 (3442.55); HR-ESI-MS (*m/z*) for $[M+3H]^{3+}$ (calculated): 1135.5407 (1135.5276).

5: ($C_{145}H_{218}N_{36}O_{39}S_3$): HPLC gradient (semipreparative): 15 – 85 % MeCN + 0.1 % TFA in 8 min, R_t = 4.00 min; 0 – 100 % MeCN + 0.1 % TFA in 12 min, R_t = 6.50 min. HPLC gradient (analytical): 0 – 100 % MeCN + 0.1 % TFA in 8 min, R_t = 3.10 min, yield: 75 %, purity: 95 %,

MALDI-MS (m/z) for $[M+H]^+$ (calculated): 3183.69 (3184.53); $[M+Na]^+$ (calculated): 3206.14 (3206.52); $[M+K]^+$ (calculated): 3222.47 (3222.51); HR-ESI-MS (*m/z*) for $[M+3H]^{3+}$ (calculated): 1062.1903 (1062.1781).

General synthesis of the multimodal-Imaging-units (MIU) 6a – 13a

Cys(Trt)-Lys(alloc)-NODA-GA(tBu)$_3$ was synthesized according to standard Fmoc-based solid phase peptide synthesis protocols.[1] The allyloxycarbonyl protecting group was removed by *tetrakis*(triphenylphosphine)palladium(0). 50 µmol Cys(Trt)-Lys-NODA-GA(tBu)$_3$ on rink amide resin (100 mg) were reacted with 4 eq. of the respective dye **6b** – **13b** (100 µmol) in DMF (4 mL) for 1 h. **6a** – **10a**: the dyes **6b** – **10b** were added without prior activation, 4 eq. of DIPEA were added after 30 minutes, **11a** – **13a**: the dyes **11b** – **13b** were activated beforehand with HBTU (0.95 eq.) and DIPEA (1.0 eq.) as base, in DMF (2 - 4 mL), for 2 minutes. After dye conjugation, the resin was filtered from solvent and washed subsequently thrice with DMF, water, dichloromethane and diethylether. After drying of the resin, the conjugates were cleaved from resin and at the same time deprotected by using a TFA/TIS (95 % / 5 %, 5 mL) mixture for one to two hour(s). The volatile materials were removed by reduced pressure and the residue was dissolved in 1:1 MeCN:H_2O + 0.1 % TFA and purified by HPLC.

Analytical Part **6a** – **13a**:

6a: ($C_{45}H_{65}N_8O_9S$)(CF_3CO_2): HPLC gradient (analytical): 0 – 100 % MeCN + 0.1 % TFA in 12 min, R_t = 5.77 min, yield: 30 %, purity: 98 %, MALDI-MS (m/z) for $[M]^+$ (calculated): 893.28 (893.46); HR-ESI-MS (*m/z*) for [M-4H+K+Na]+ (calculated): 951.3426 (951.3811).

7a: ($C_{41}H_{61}N_8O_9S$)(CF_3CO_2): HPLC gradient (analytical): 0 – 100 % MeCN + 0.1 % TFA in 12 min, R_t = 5.83 min, yield: 30 %, purity: 98 %, MALDI-MS (m/z) for $[M]^+$ (calculated): 841.25 (841.43); HR-ESI-MS (*m/z*) for [M-2H+K+Na]+ (calculated): 901.3257 (901.3655).

8a: ($C_{43}H_{63}N_8O_9S$)(CF_3CO_2): HPLC gradient (analytical): 0 – 100 % MeCN + 0.1 % TFA in 12 min, R_t = 7.67 min, yield: 30 %, purity: 98 %, MALDI-MS (m/z) for $[M]^+$ (calculated): 867.15 (867.44); HR-ESI-MS (*m/z*) for [M-2H+K+Na]+ (calculated): 927.3420 (927.3811).

9a: ($C_{36}H_{54}N_8O_{11}S_2$): HPLC gradient (analytical): 0 – 100 % MeCN + 0.1 % TFA in 12 min, R_t = 5.25 min, yield: 35 %, purity: 85 %, MALDI-MS (m/z) for $[M+H]^+$ (calculated): 839.48 (839.34); $[M+Na]^+$ (calculated): 861.49 (861.33); $[M+K]^+$ (calculated): 877.50 (877.30); HR-ESI-MS (*m/z*) $[M-H+K+Na]^+$ (calculated): 899.2405 (899.3353).

10a: ($C_{30}H_{44}N_{10}O_{12}S$): HPLC gradient (analytical): 0 – 100 % MeCN + 0.1 % TFA in 12 min, R_t = 5.75 min, yield: 20 %, purity: 99 %, MALDI-MS (m/z) for $[M+H]^+$ (calculated): 769.43 (769.29); $[M+Na]^+$ (calculated): 791.45 (791.28); $[M+K]^+$ (calculated): 807.49 (807.25); HR-ESI-MS (*m/z*) for $[M-H+K+Na]^+$ (calculated): 829.1922 (829.2861).

11a: ($C_{45}H_{53}N_9O_7S$): HPLC gradient (analytical): 0 – 100 % MeCN + 0.1 % TFA in 12 min, R_t = 5.80 min, yield: 30 %, purity: 97 %, MALDI-MS (m/z) for $[M+H]^+$ (calculated): 964.26 (964.34);

$[M+Na]^+$ (calculated): 986.28 (986.32); $[M+K]^+$ (calculated): 1002.25 (1002.30); HR-ESI-MS (*m/z*) for $[M-H+K+Na]^+$ (calculated): 1024.2365 (1024.2777).

12a: ($C_{40}H_{56}N_8O_{12}S$): HPLC gradient (analytical): 0 – 100 % MeCN + 0.1 % TFA in 12 min, R_t = 6.36 min, yield: 25 %, purity: 97 %, MALDI-MS (m/z) for $[M+H]^+$ (calculated): 873.09 (873.38); $[M+Na]^+$ (calculated): 895.11 (895.36); $[M+K]^+$ (calculated): 911.09 (911.34); HR-ESI-MS (*m/z*) for $[M-H+K+Na]^+$ (calculated): 933.2790 (933.3195).

13a: ($C_{56}H_{72}N_9O_{11}S$)(CF_3CO_2): HPLC gradient (analytical): 0 – 100 % MeCN + 0.1 % TFA in 12 min, R_t = 7.77 min, yield: 30 %, purity: 95 %, MALDI-MS (m/z) for $[M+H]^+$ (calculated): 1078.08 (1078.51); $[M+Na]^+$ (calculated): 1100.04 (1100.49)); $[M+K]^+$ (calculated): 1116.00 (1116.46); HR-ESI-MS (*m/z*) for $[M-2H+K+Na]^+$ (calculated): 1138.4063 (1138.4445).

General Synthesis of dimeric-PESIN-MIU-conjugates 6 – 13 and dimeric-PESIN-S$^{t\text{-}}$*Bu* 14

1.17 µmol of **5** (4 mg) and 1.3 µmol of **6a – 14a** were dissolved in 1:1 MeCN:H_2O + 0.1 % TFA and pH = 7 was adjusted with 0.5 M phosphate buffer (pH = 7.2), 5 minutes later followed by HPLC purification to give the colored solids **6 – 8**, **10 - 13** or the white powders **9**, **14** respectively.

Analytical Part **6 - 14**:
6: ($C_{200}H_{291}N_{46}O_{52}S_4$)($CF_3CO_2$): HPLC gradient: 0 – 100 % MeCN + 0.1 % TFA in 12 min, R_t = 7.00 min, yield: 35 %, purity: 99 %, MALDI-MS (m/z) for $[M]^+$ (calculated): 4297.72 (4297.04); $[M+Na]^+$ (calculated): 4320.04 (4319.02); $[M+K]^+$ (calculated): 4335.61 (4336.01); $[M+H]^{2+}$ (calculated): 2149.03 (2149.02) HR-ESI-MS (*m/z*) for $[M+3Na]^{4+}$ (calculated): 1091.5015 (1091.5028).

7: ($C_{196}H_{287}N_{46}O_{52}S_4$)($CF_3CO_2$): HPLC gradient: 0 – 100 % MeCN + 0.1 % TFA in 12 min, R_t = 7.00 min, yield: 75 %, purity: 99 %, MALDI-MS (m/z) for $[M]^+$ (calculated): 4245.85 (4245.01); $[M+Na]^+$ (calculated): 4267.19 (4266.99); $[M+K]^+$ (calculated): 4283.11 (4282.97); $[M+H]^{2+}$ (calculated) : 2123.20 (2123.01); HR-ESI-MS (*m/z*) for $[M+3Na]^{4+}$ (calculated): 1078.4934 (1078.4950).

8: ($C_{198}H_{289}N_{46}O_{52}S_4$)($CF_3CO_2$): HPLC gradient: 0 – 100 % MeCN + 0.1 % TFA in 12 min, R_t = 5.65 min, yield: 85 %, purity: 99 %, MALDI-MS (m/z) for $[M]^+$ (calculated): 4271.43 (4271.03); $[M+Na]^+$ (calculated): 4293.87 (4293.01); $[M+K]^+$ (calculated): 4308.09 (4308.98); $[M+3Na]^+$ (calculated): 4335.80 (4336.97); $[M+H]^{2+}$ (calculated): 2136.04 (2136.02); HR-ESI-MS (*m/z*) for $[M+3Na]^{4+}$ (calculated): 1084.9973 (1084.9989).

9: ($C_{191}H_{280}N_{46}O_{54}S_5$): HPLC gradient: 0 – 100 % MeCN + 0.1 % TFA in 12 min, R_t = 6.40 min, yield: 85 %, purity: 98 %, MALDI-MS (m/z) for $[M+H]^+$ (calculated): 4242.80 (4242.93); $[M+Na]^+$ (calculated): 4265.08 (4264.91); $[M+K]^+$ (calculated): 4280.56 (4279.87); HR-ESI-MS (*m/z*) for $[M+3Na]^{3+}$ (calculated): 1436.2970 (1436.2906).

10: ($C_{185}H_{270}N_{48}O_{55}S_4$): HPLC gradient: 0 – 100 % MeCN + 0.1 % TFA in 12 min, R_t = 6.50 min, yield: 65 %, purity: 98 %, MALDI-MS (m/z) for $[M+H]^+$ (calculated): 4172.53 (4172.88); $[M+Na]^+$ (calculated): 4194.67 (4194.86); $[M+K]^+$ (calculated): 4211.33 (4210.83); HR-ESI-MS (*m/z*) for $[M-H+3Na+K]^{3+}$ (calculated): 1426.2575 (1426.2647).

11: ($C_{200}H_{279}N_{45}O_{58}S_4$): HPLC gradient: 0 – 100 % MeCN + 0.1 % TFA in 12 min, R_t = 6.50 min, yield: 30 %, purity: 99 %, MALDI-MS (m/z) for $[M+H]^+$ (calculated): 4368.06 (4367.92); $[M+Na]^+$ (calculated): 4390.68 (4389.90); $[M+K]^+$ (calculated): 4406.18 (4405.89); HR-ESI-MS (*m/z*) for $[M+H+3Na]^{4+}$ (calculated): 1109.2219 (1109.2230).

12: ($C_{195}H_{282}N_{46}O_{55}S_4$): HPLC gradient: 0 – 100 % MeCN + 0.1 % TFA in 12 min, R_t = 6.85 min, yield: 80 %, purity: 99 %, MALDI-MS (m/z) for $[M+H]^+$ (calculated): 4276.97 (4276.96); $[M+Na]^+$ (calculated): 4298.91 (4298.95); $[M+K]^+$ (calculated): 4314.54 (4314.92); $[M+2H]^{2+}$ (calculated): 2138.64 (2138.99); HR-ESI-MS: This compound could not be detected via ESI-MS.

13: ($C_{211}H_{298}N_{47}O_{54}S_4$)($CF_3CO_2$): HPLC gradient: 0 – 100 % MeCN + 0.1 % TFA in 12 min, R_t = 7.35 min, yield: 65 %, purity: 99 %, MALDI-MS (m/z) for $[M]^+$ (calculated): 4482.20 (4482.09); $[M+Na]^+$ (calculated): 4504.07 (4504.07); $[M+K]^+$ (calculated): 4520.15 (4520.05); $[M+2H]^{2+}$ (calculated): 2240.83 (2241.55); HR-ESI-MS (*m/z*) for $[M+2Na+K]^{4+}$ (calculated): 1141.7629 (1141.7582).

14: ($C_{159}H_{236}N_{38}O_{43}S_4$): HPLC gradient: 0 – 100 % MeCN + 0.1 % TFA in 12 min, R_t = 6.80 min, yield: 60 %, purity: 98 %, MALDI-MS (m/z) for $[M+H]^+$ (calculated): 3494.70 (3494.64); $[M+Na]^+$ (calculated): 3516.92 (3516.62); $[M+K]^+$ (calculated): 3532.73 (3532.60); $[M+2H]^{2+}$ (calculated): 1748.15 (1747.82); ESI-MS (*m/z*) for $[M+3H]^{3+}$ (calculated): 1165.5580 (1165.5443).

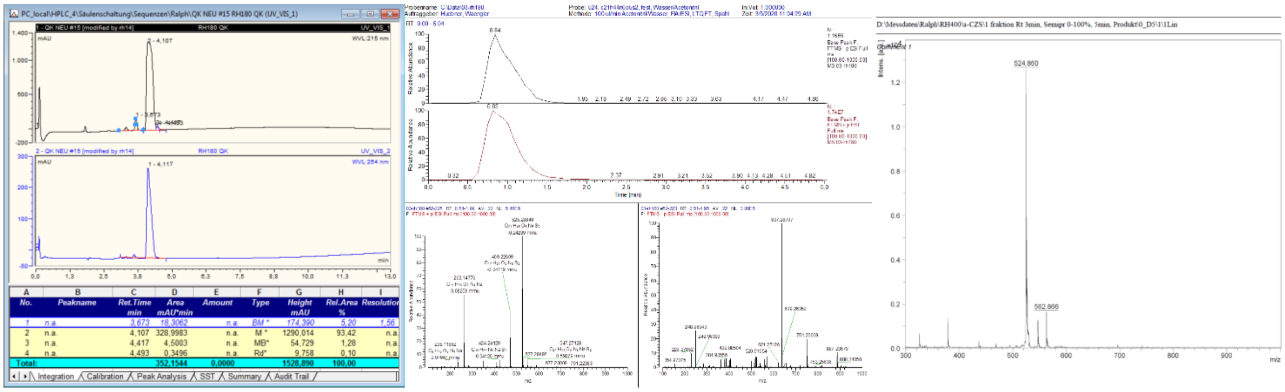

Fig. S1 Analytical data for **1** (HPLC, ESI and MALDI mass spectroscopy).

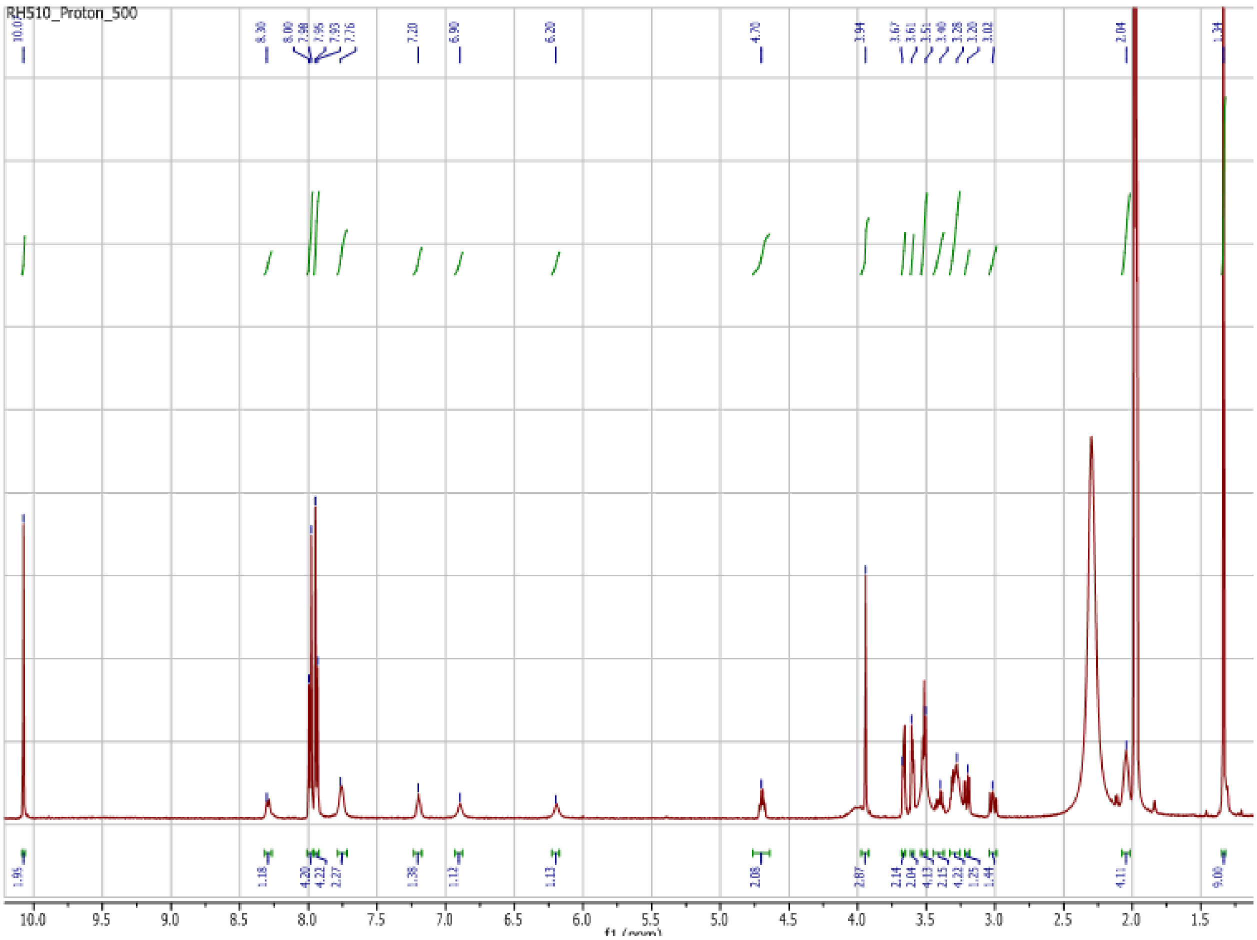

Fig. S2 ^{1}H-NMR of **1** (CD_3CN, 400 MHz).

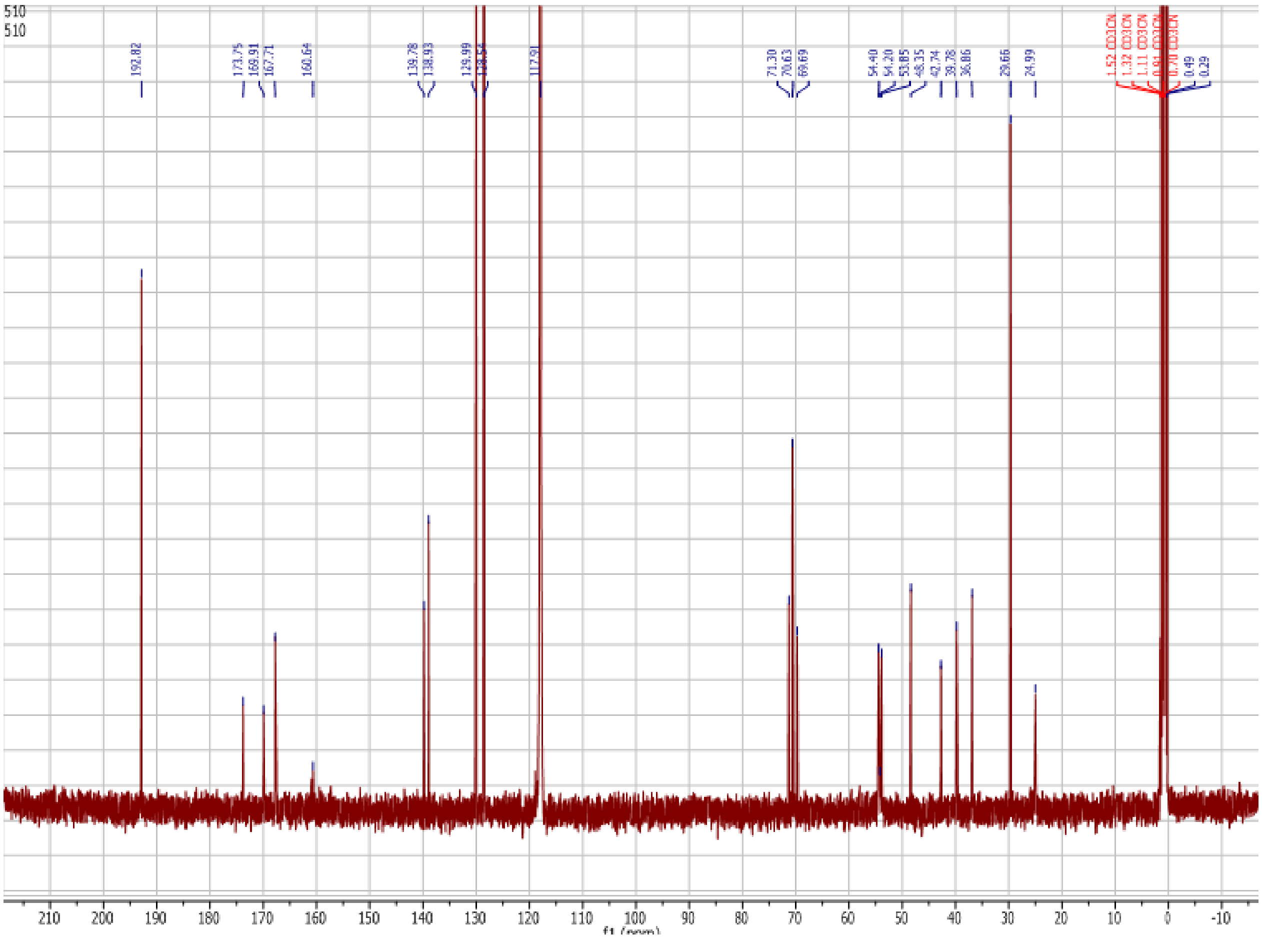

Fig. S3 ^{13}C-NMR of **1** (CD_3CN, 101 MHz).

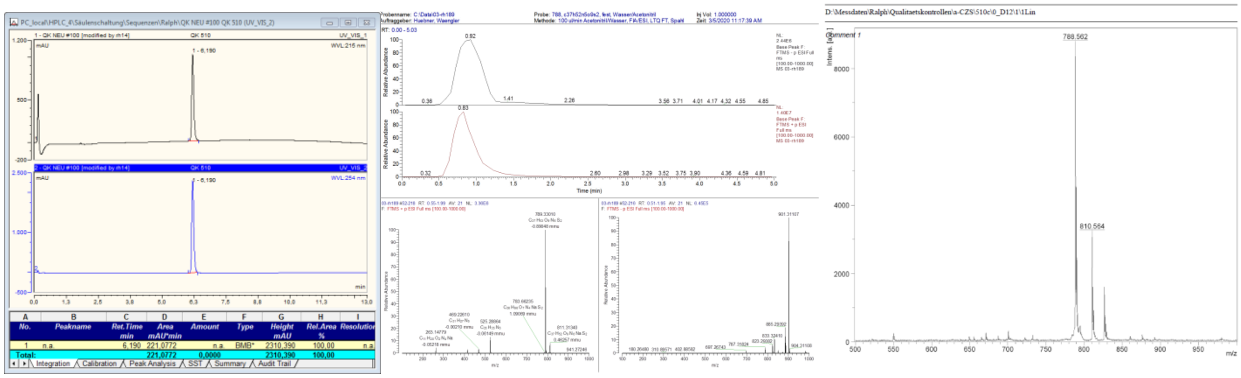

Fig. S4 Analytical data for **2** (HPLC, ESI and MALDI mass spectroscopy).

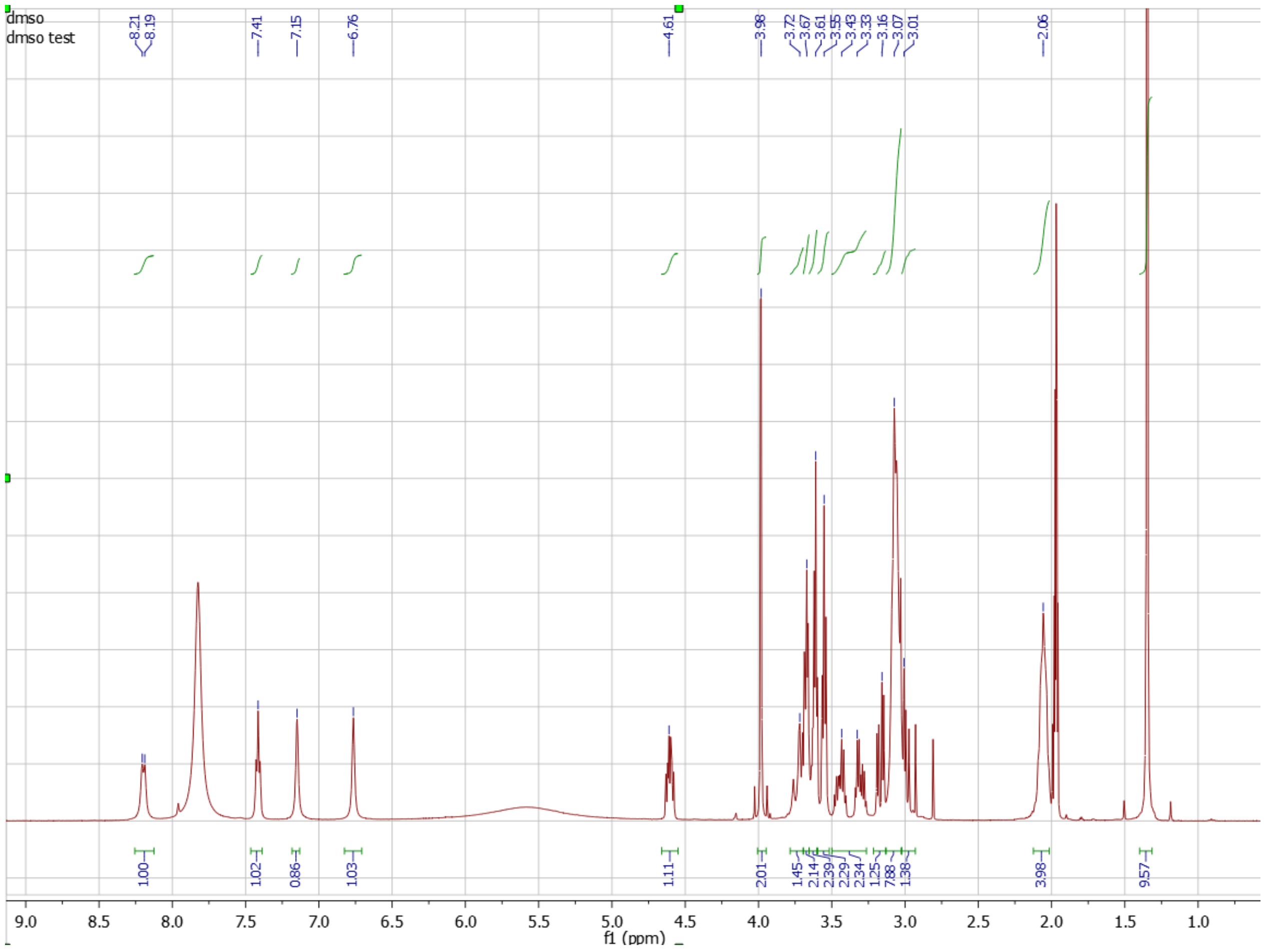

Fig. S5 ^{1}H-NMR of **2** (CD_3CN, 400 MHz).

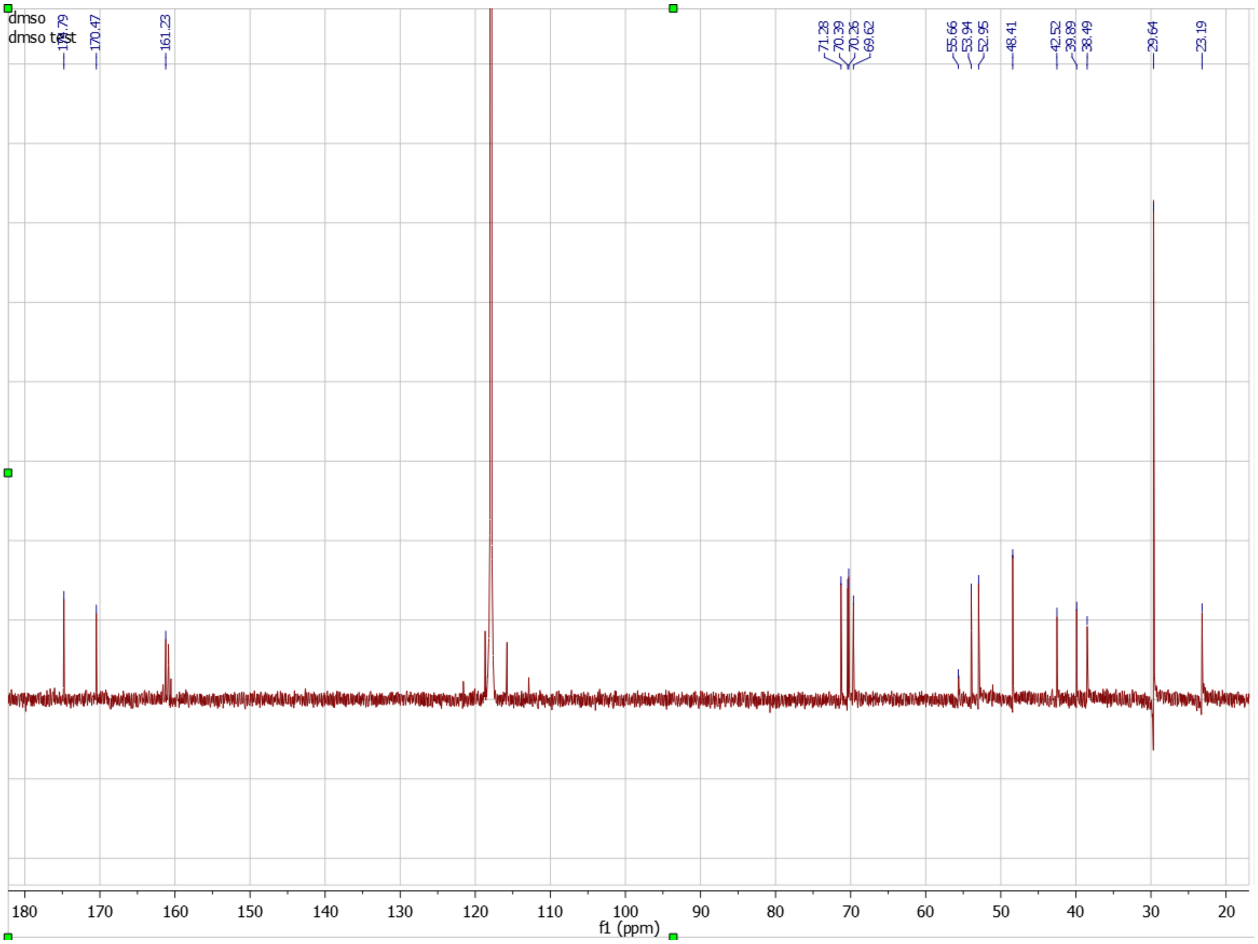

Fig. S6 ^{13}C-NMR of **2** (CD_3CN, 101 MHz).

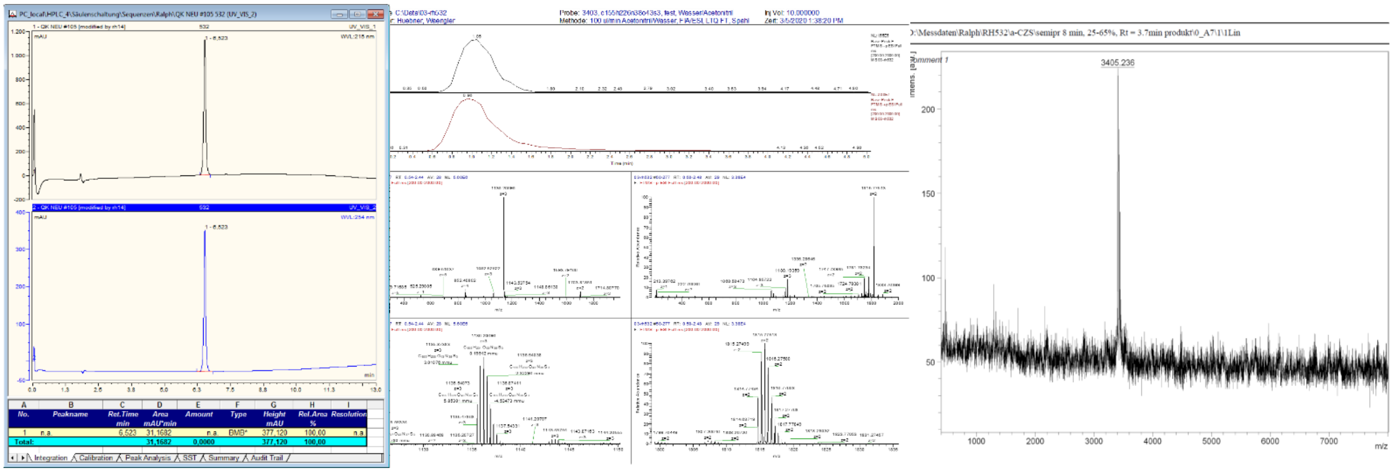

Fig. S7 Analytical data for **4** (HPLC, ESI and MALDI mass spectroscopy).

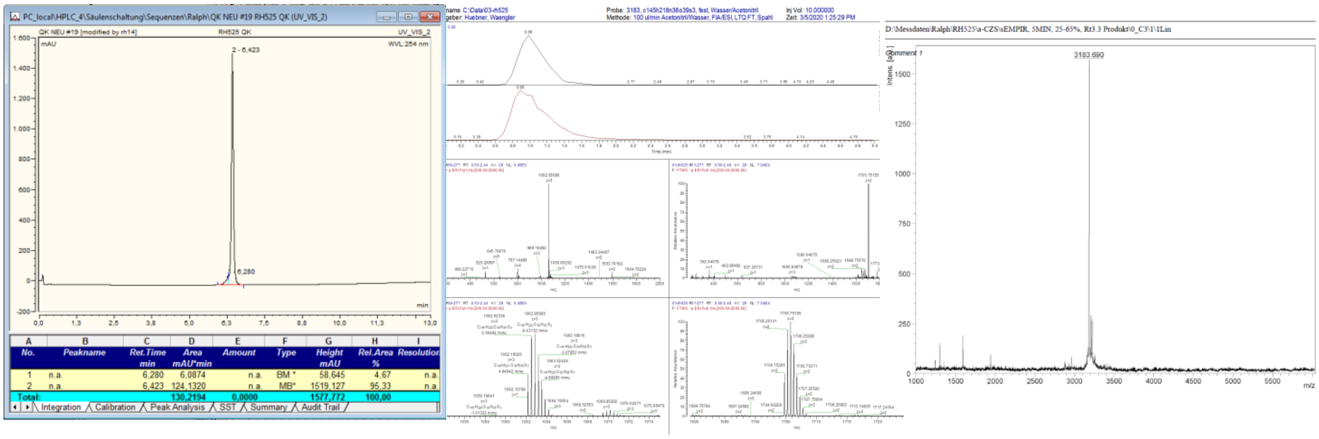

Fig. S8 Analytical data for **5** (HPLC, ESI and MALDI mass spectroscopy).

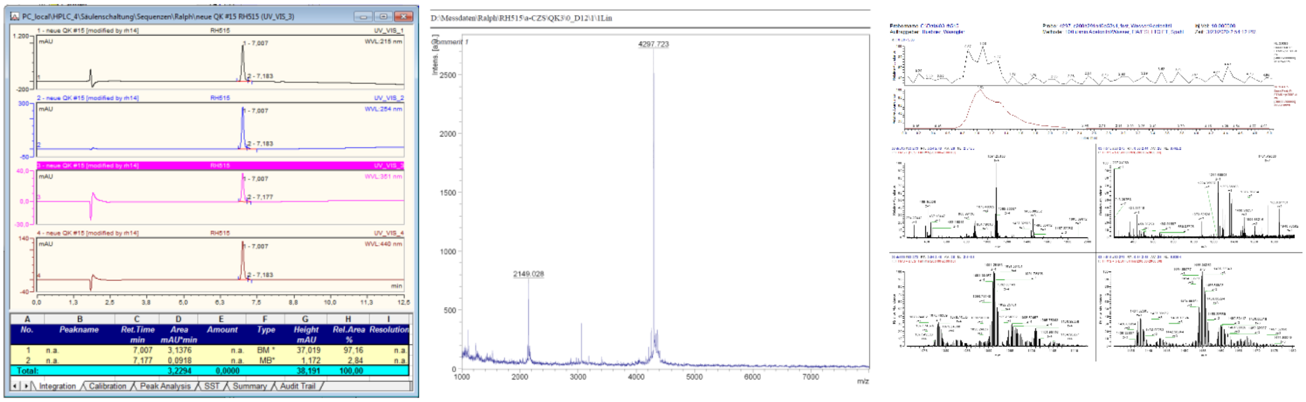

Fig. S9 Analytical data for **6** (HPLC, MALDI and ESI mass spectroscopy).

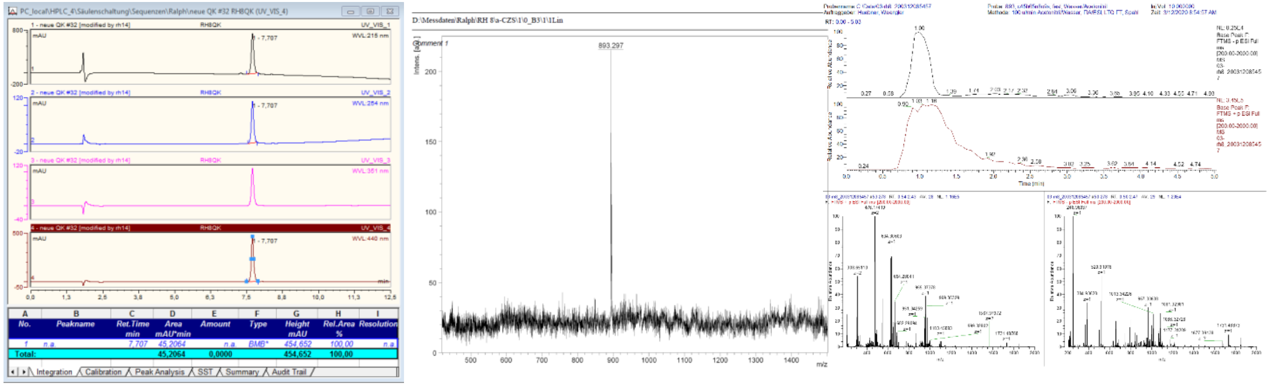

Fig. S10 Analytical data for **6a** (HPLC, MALDI and ESI mass spectroscopy).

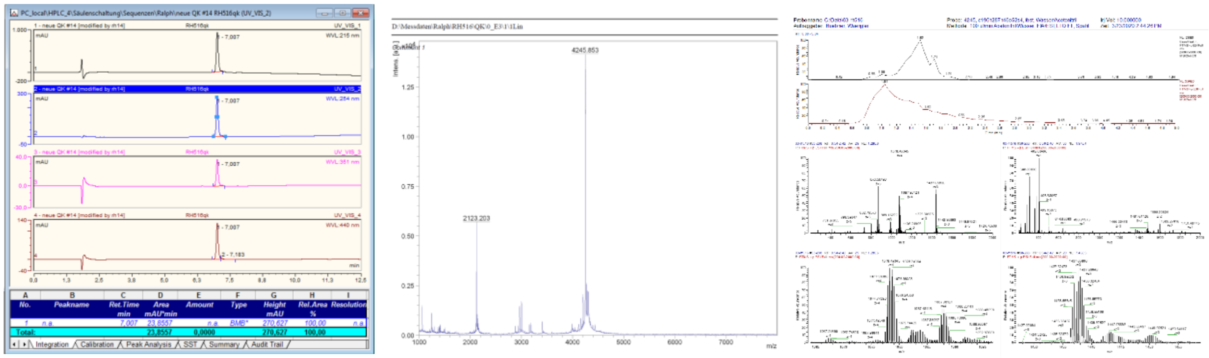

Fig. S11 Analytical data for **7** (HPLC, MALDI and ESI mass spectroscopy).

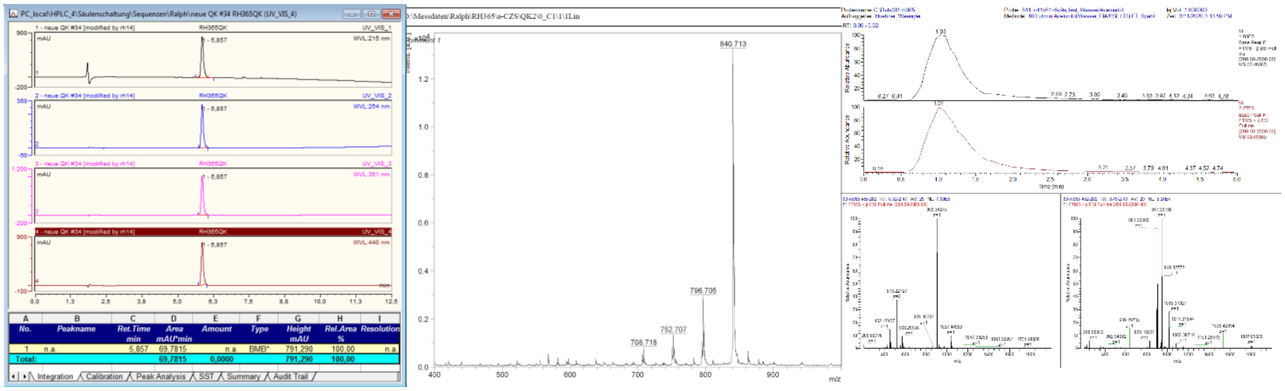

Fig. S12 Analytical data for **7a** (HPLC, MALDI and ESI mass spectroscopy).

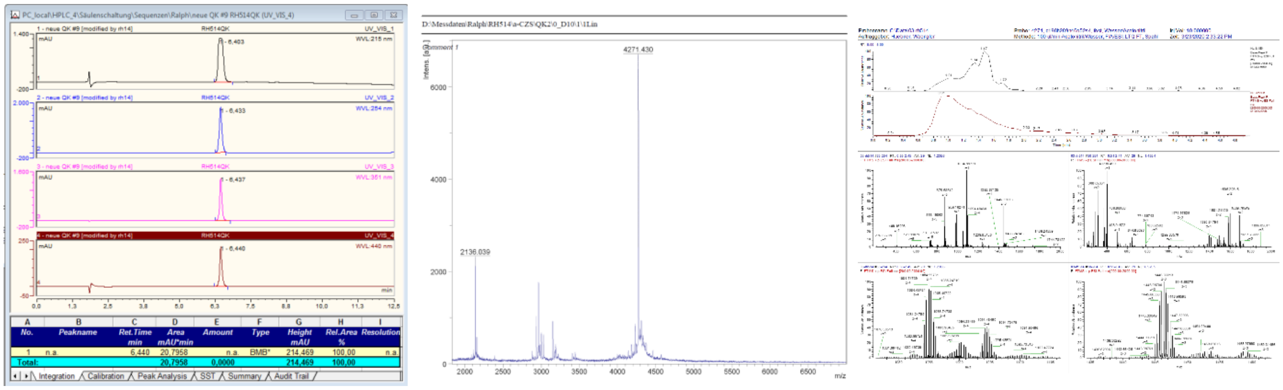

Fig. S13 Analytical data for **8** (HPLC, MALDI and ESI mass spectroscopy).

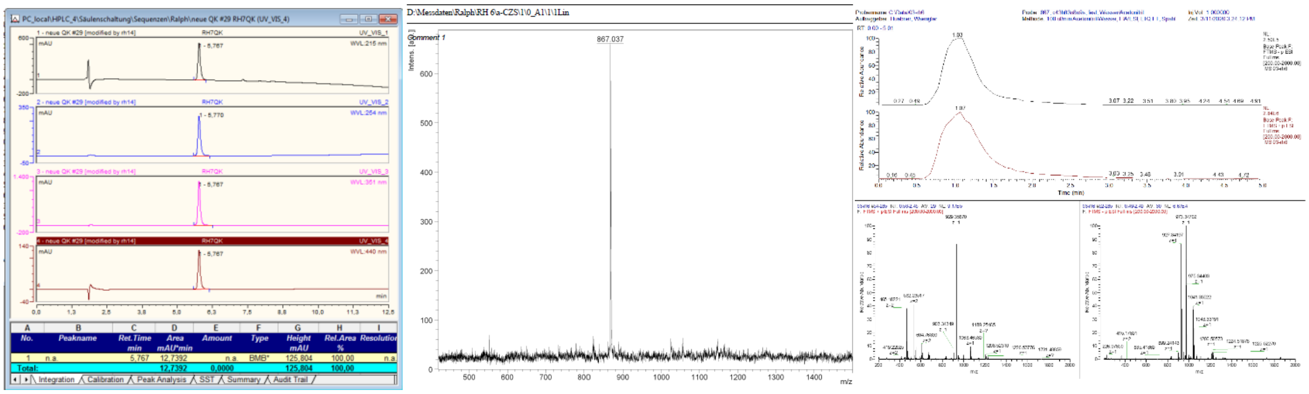

Fig. S14 Analytical data for **8a** (HPLC, MALDI and ESI mass spectroscopy).

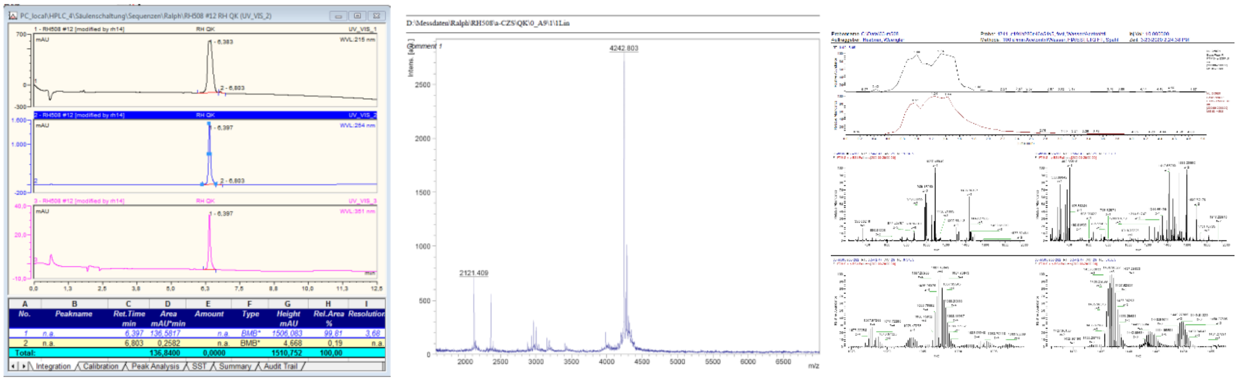

Fig. S15 Analytical data for **9** (HPLC, MALDI and ESI mass spectroscopy).

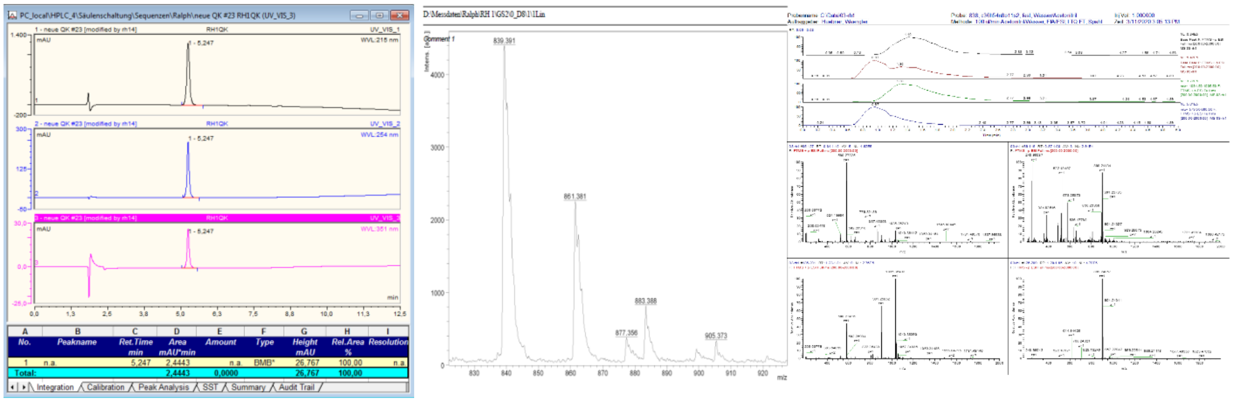

Fig. S16 Analytical data for **9a** (HPLC, MALDI and ESI mass spectroscopy).

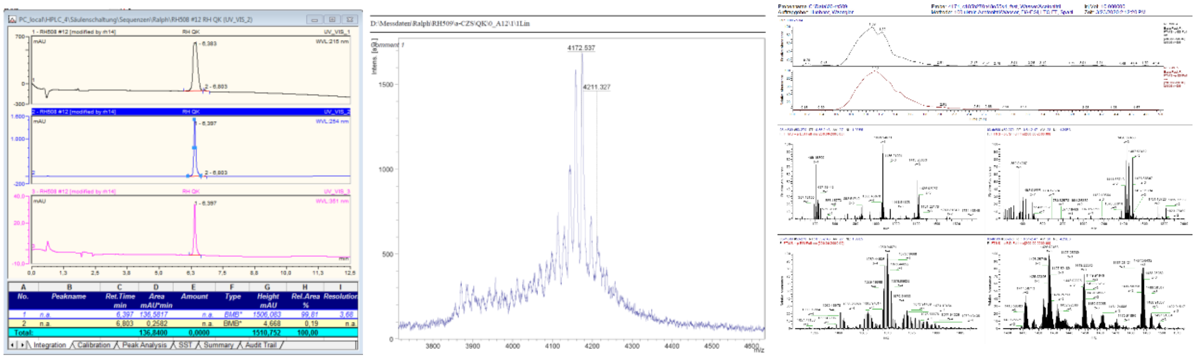

Fig. S17 Analytical data for **10** (HPLC, MALDI and ESI mass spectroscopy).

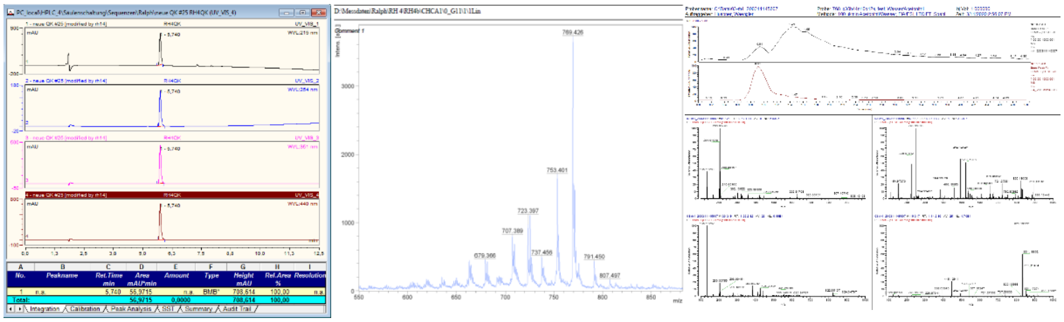

Fig. S18 Analytical data for **10a** (HPLC, MALDI and ESI mass spectroscopy).

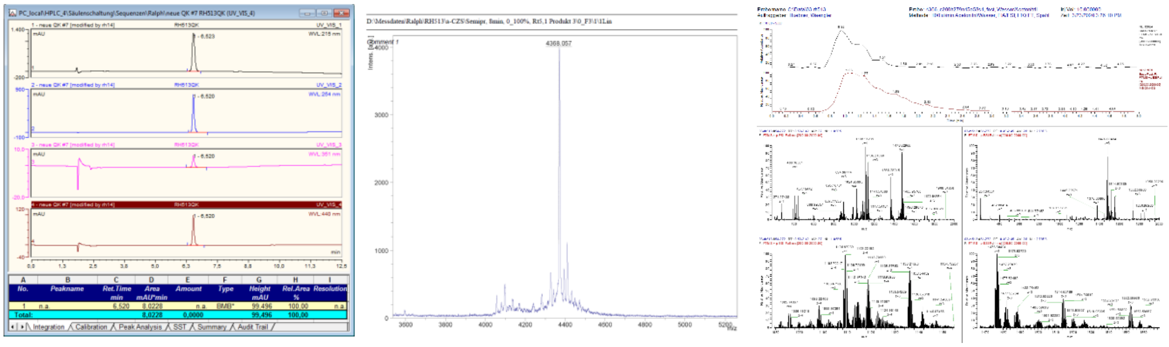

Fig. S19 Analytical data for **11** (HPLC, MALDI and ESI mass spectroscopy).

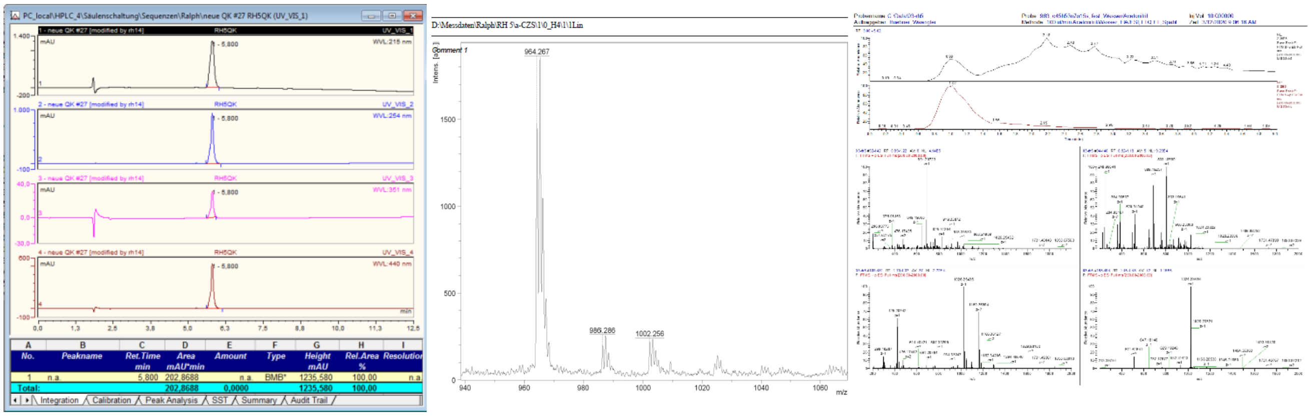

Fig. S20 Analytical data for **11a** (HPLC, MALDI and ESI mass spectroscopy).

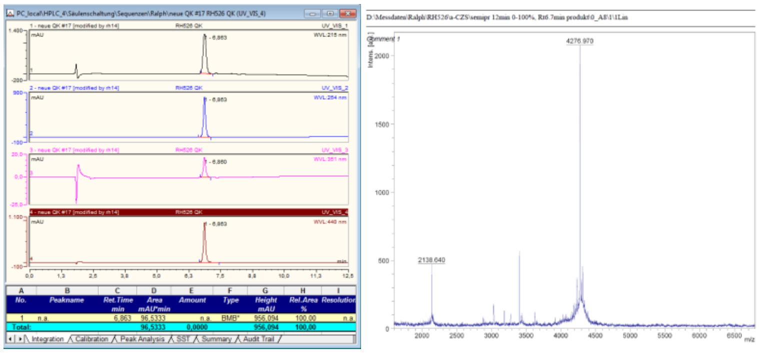

Fig. S21 Analytical data for **12** (HPLC and MALDI mass spectroscopy).

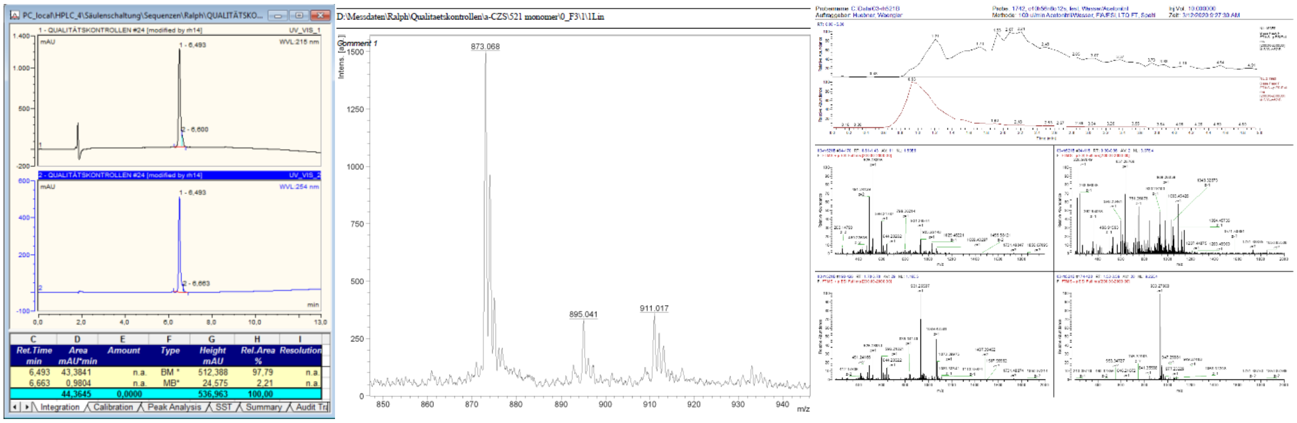

Fig. S22 Analytical data for **12a** (HPLC, MALDI and ESI mass spectroscopy).

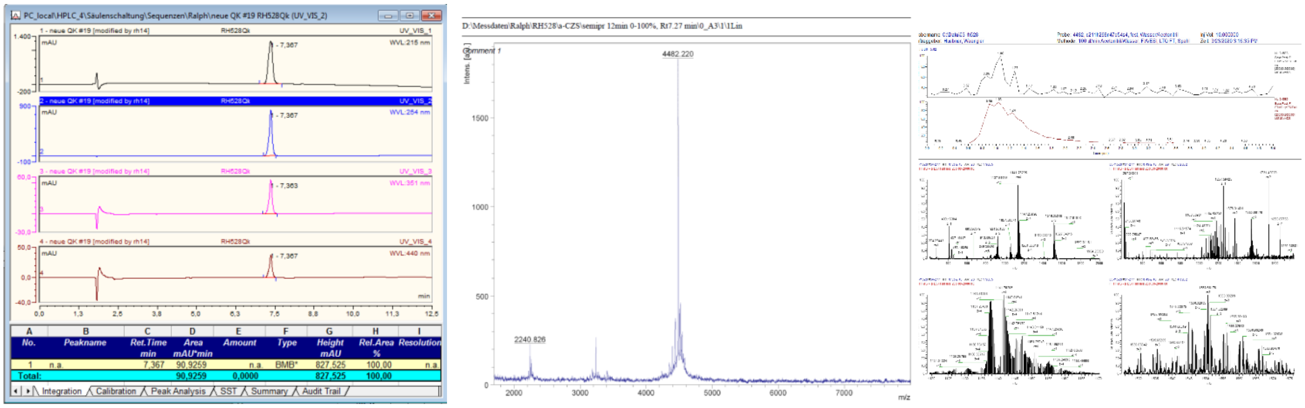

Fig. S23 Analytical data for **13** (HPLC, MALDI and ESI mass spectroscopy).

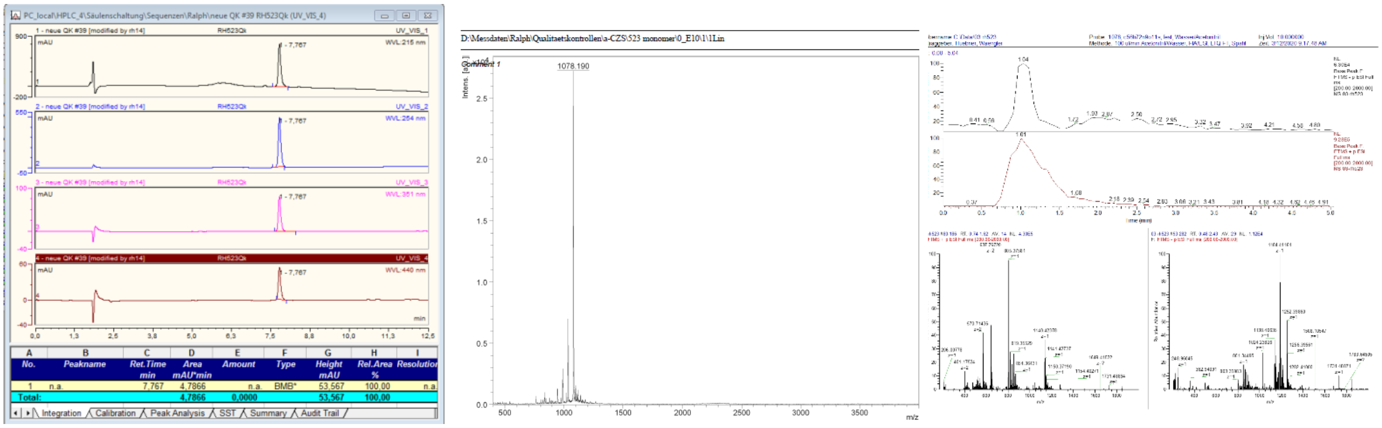

Fig. S24 Analytical data for **13a** (HPLC, MALDI and ESI mass spectroscopy).

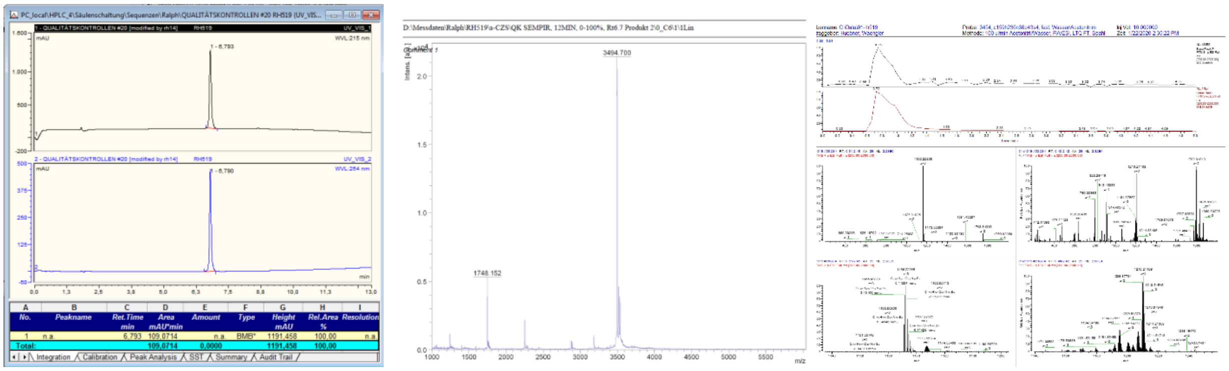

Fig. S25 Analytical data for **14** (HPLC, MALDI and ESI mass spectroscopy).

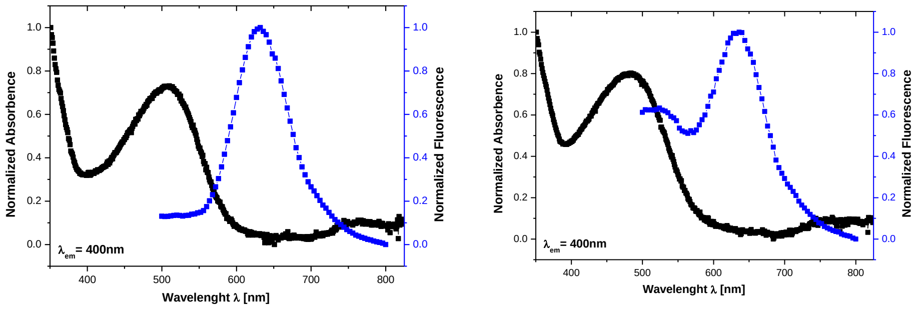

Fig. S26 Normalized absorption and emission spectra of **6** (left) and **6a** (right), recorded in PBS at a concentration of c = 1.0 x 10^{-5} mol/L, emission wavelength stated in the graph (bottom, left corner).

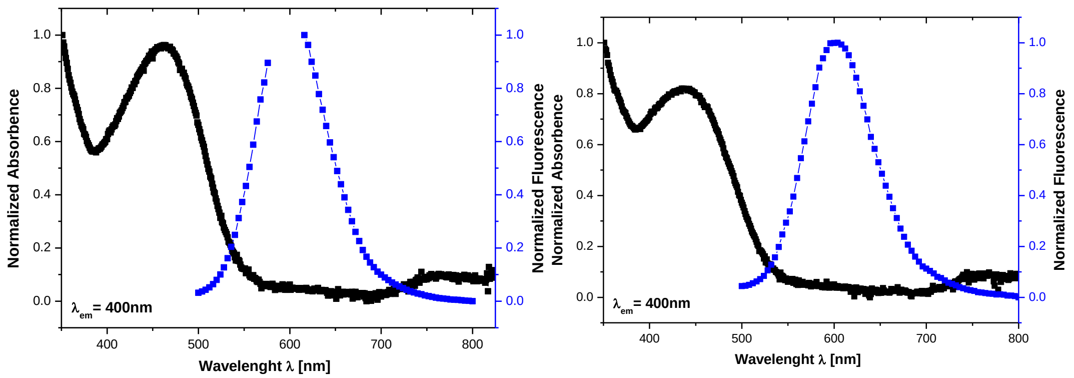

Fig. S27 Normalized absorption and emission spectra of **7** (left) and **7a** (right), recorded in PBS at a concentration of c = 1.0 x 10^{-5} mol/L, emission wavelength stated in the graph (bottom, left corner).

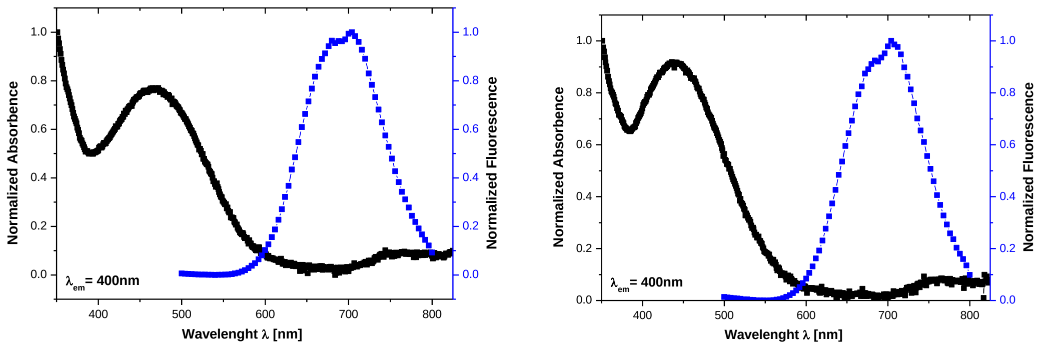

Fig. S28 Normalized absorption and emission spectra of **8** (left) and **8a** (right), recorded in PBS at a concentration of c = 1.0 x 10^{-5} mol/L, emission wavelength stated in the graph (bottom, left corner).

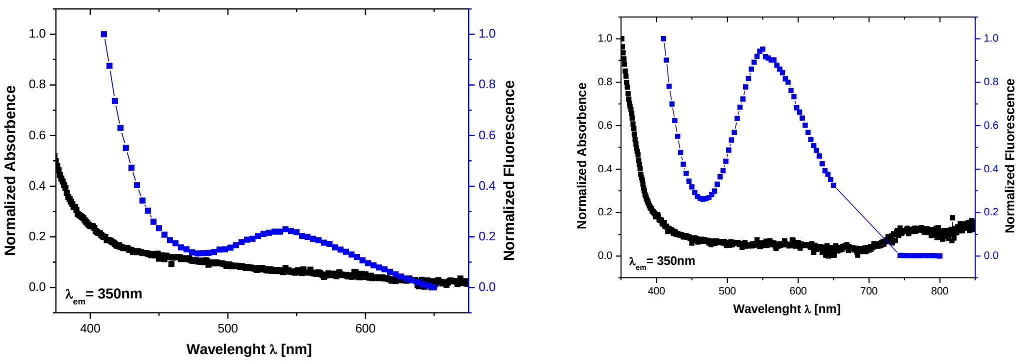

Fig. S29 Normalized absorption and emission spectra of **9** (left) and **9a** (right), recorded in PBS at a concentration of c = 1.0 x 10^{-5} mol/L, emission wavelength stated in the graph (bottom, left corner).

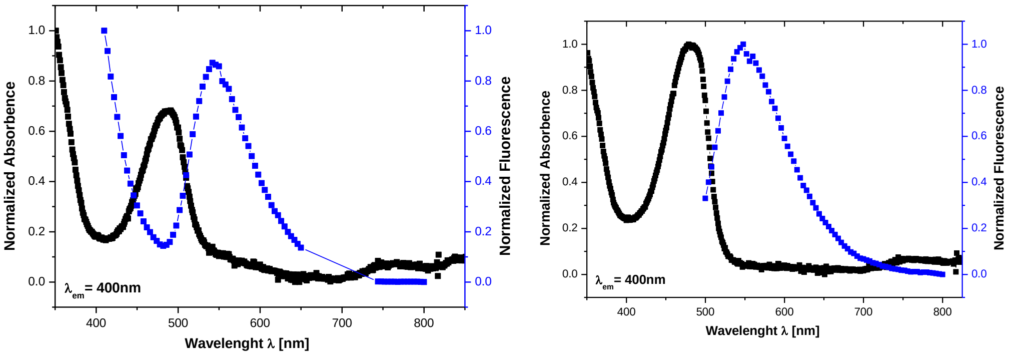

Fig. S30 Normalized absorption and emission spectra of **10** (left) and **10a** (right), recorded in PBS at a concentration of c = 1.0 x 10^{-5} mol/L, emission wavelength stated in the graph (bottom, left corner).

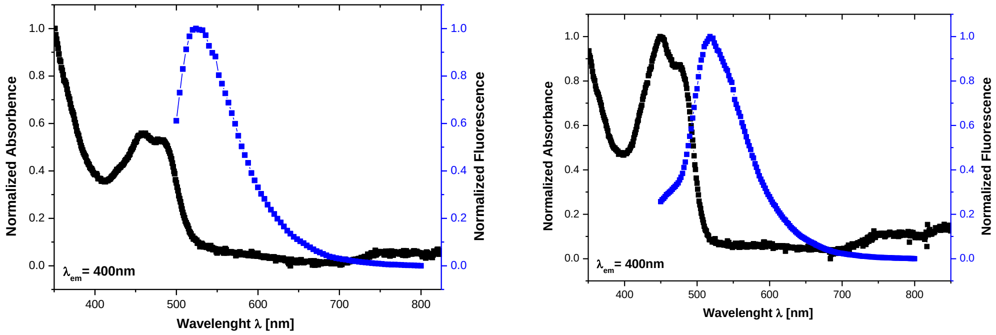

Fig. S31 Normalized absorption and emission spectra of **11** (left) and **11a** (right), recorded in PBS at a concentration of c = 1.0 x 10^{-5} mol/L, emission wavelength stated in the graph (bottom, left corner).

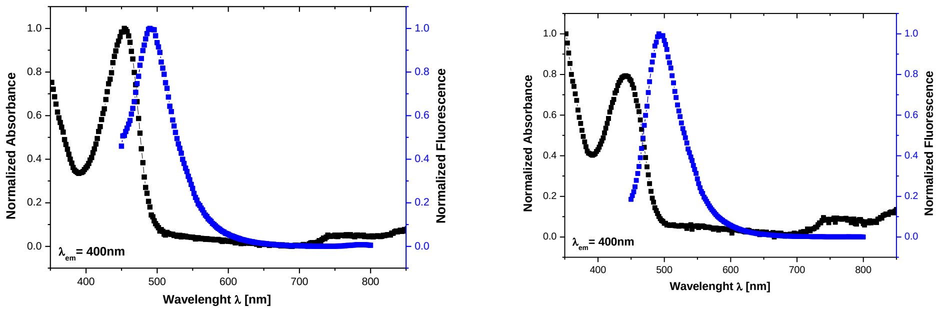

Fig. S32 Normalized absorption and emission spectra of **12** (left) and **12a** (right), recorded in PBS at a concentration of c = 1.0 x 10^{-5} mol/L, emission wavelength stated in the graph (bottom, left corner).

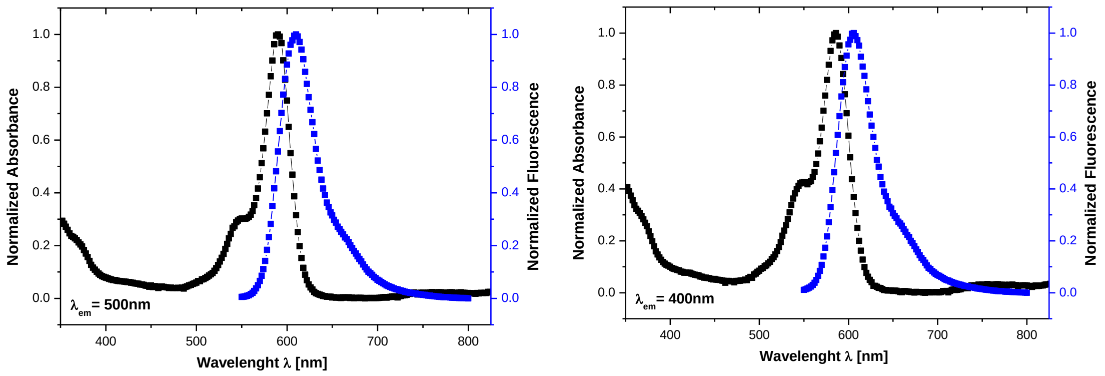

Fig. S33 Normalized absorption and emission spectra of **13** (left) and **13a** (right), recorded in PBS at a concentration of c = 1.0 x 10^{-5} mol/L, emission wavelength stated in the graph (bottom, left corner).

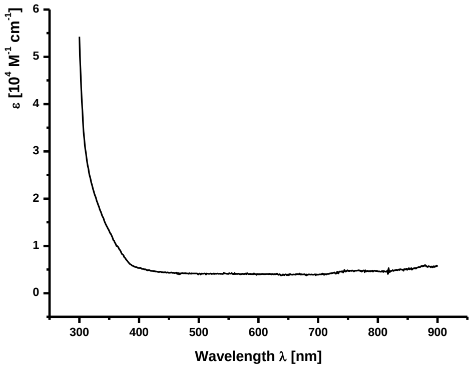

Fig. S34 Absorption spectrum of **14,** recorded in PBS at a concentration of c = 1.0 x 10^{-5} mol/L.

Radiochemistry

A solution of the respective multimodal imaging unit (MIU) **6a – 13a** (5 nmol) or multimodal imaging agent **6** - **14** (5 nmol) in H_2O (Tracepur quality, 1mM) was added to 90 - 120 MBq of [^{68}Ga]$GaCl_3$ in a solution obtained by fractioned elution of an IGG ^{68}Ge/^{68}Ga generator system with HCl (0.1 M, 1.6 mL) and subsequent titration to pH 3.5 – 4.2 by addition of sodium acetate solution (1.25 M, 50 – 75 µL). All labeling experiments were performed by addition of 2 mg ascorbic acid to suppress radiolysis-induced product fragmentation. In the labeling experiments of the MIUs **6a – 13a**, 1 - 4 mg TCEP x HCl were added and the pH was adjusted to 3.5 – 4.2 by addition of sodium acetate solution (1.25 M). After reaction for 10 min at 45°C, the reaction mixtures were analyzed by analytical radio-HPLC. The radiolabeled products were found to be 95 – 99 % pure and obtained in non-optimized molar activities of 90 - 120 GBq/µmol.

Log$_D$ determination

For ^{68}Ga-**6/6a** – ^{68}Ga-**13/13a**, the water/1-octanol partition coefficient (log$_D$) was determined by adding 5 µL of the respectively ^{68}Ga-labeled compound (0.8 – 1.2 MBq) to a mixture of phosphate buffer (0.05 M, pH 7.4, 795 µL) and 1-octanol (800 µL). The mixtures were intensively shaken for 5 minutes on a vibrating plate. After subsequent centrifugation at 13 000 rpm for 5 min, 125 µL were taken from each phase and measured in a γ-counter. The log$_D$ values were calculated from three or four independent experiments, each performed in triplicate.

For **14**, the water/1-octanol partition coefficient (log$_D$) was determined by semipreparative HPLC. For this purpose, 10 µL solution (c = 5 x 10^{-4} mol/L) of the substance were added to a mixture of 1 mL 1-octanol and 990 µL phosphate buffered solution (0.05 M, pH 7.4) and the mixture was vigorously shaken for 5 minutes. After centrifugation, the phases were separated and both phases were analyzed by semipreparative HPLC. The log$_D$ was determined by three separate measurements, each experiment performed in triplicate.

Competitive receptor binding assay

Stably GRPR-transfected Human Embryonic Kidney 293 cells (HEK-GRPR) were cultured at 37°C in Dulbecco's Modified Eagle's Medium (DMEM, high glucose, GlutaMax-I, 500 mL) supplemented with 10 % FCS (50 mL), 1.5 % Geniticin (8.25 mL) and 1 % PenStrep (5.5 mL) in a humidified atmosphere containing 5 % CO_2. The medium was exchanged every two or three days and cells were split at >75 % confluence. *In vitro* binding affinities were determined via competitive displacement experiments which were performed at least trice, each experiment performed in triplicate. A Millipore Multiscreen punch kit and Millipore 96 well filter plates (pore size 1.2 µm) were used. The plates were incubated with PBS/BSA (1%) solution (each well 200 µL) for one hour before use. HEK-GRPR cells were harvested and suspended carefully in Opti-MEM I (GlutaMAX I) medium. 50 µL of a cell suspension containing 10^5 cells were seeded in each well. To this, a total volume of 50 µL was added to each well, containing 25 µL (0.012 kBq/µL) of the GRPR-specific radioligand [^{125}I]-Tyr^4-bombesin (81.4 GBq/µmol) and 25 µL of the respective competitor **6** – **14** or endogenous bombesin (BBN, used as reference compound). The competitor was added in 11 increasing concentrations ranging from 0.25 – 500 nM for **6** –

14 or 0.1 – 250 nM for BBN, whereat the twelfth well contained no competitor to ensure 100 % binding of the radioligand. After one hour of incubation at ambient temperature, the solution was filtrated and the filters were washed with cold PBS (3 times). The filters were collected and measured by γ-counting. The 50 % inhibitory concentration (IC_{50}) values of **6 – 14** and bombesin were calculated by fitting the obtained data via a nonlinear regression analysis using GraphPad Prism Software (v5.04).

Confocal Fluorescence Microscopy

Since the HEK-GRPR do not exhibit any adhered characteristics to glass plates, the GRPR-positive human tumor cell line PC-3 was used for the initial confocal fluorescence microscopy experiments instead. PC-3 cells were cultured at 37°C in RPMI 1640 medium supplemented with 10% FCS, 1% L-Glutamine and 1% PenStrep in a humidified atmosphere containing 5% CO_2. The medium was exchanged every two or three days and cells were split at >75% confluence. Fluorescence microscopy was performed on a Leica TCS SP8 confocal microscope with laser excitation wavelengths of 405 and 638 nm. Overlays of microscopies were generated directly with the operating software or afterwards using FIJI software (v1.50e). PC-3 cells (4×10^4) were seeded two days prior to the measurements in six-well culture plates, each well was equipped with cover slips for microscopy. Medium was exchanged after 24h. After 2 days, the medium was removed and the cells were washed carefully with PBS. Then, 1 mL of a 10 µM solution of **6** in antibiotic-free medium was added, followed by incubation for 4h. After washing of each cover slip with PBS thrice, they were consequently put reversed on object slides which were before prepared with a drop (10µL) of DAPI antifade solution and then the respective cover slide was sealed and fixed with clear coat on the object slides.

References

[1] a) D. A. Wellings and E. Atherton, *Methods in Enzymology* **1997**, *289*, 44 - 67; b) C. Wängler, S. Maschauer, O. Prante, M. Schäfer, R. Schirrmacher, P. Bartenstein, M. Eisenhut and B. Wängler, *ChemBioChem* **2010**, *11*, 2168 - 2181; c) C. Wängler, B. Waser, A. Alke, L. Iovkova, H.-G. Buchholz, S. Niedermoser, K. Jurkschat, C. Fottner, P. Bartenstein, R. Schirrmacher, J.-C. Reubi, H.-J. Wester and B. Wängler*, *2010* **2010**, *21*, 2289 - 2296.
[2] a) M. Matsui, K. Ooiwa, A. Okada, Y. Kubota, K. Funabiki and H. Sato, *Dyes and Pigments* **2013**, *99*, 916-923; b) V. G. Machado, R. I. Stock and C. Reichardt, *Chem Rev* **2014**, *114*, 10429-10475; c) Y. J. Lu, D. P. Hu, K. Zhang, W. L. Wong and C. F. Chow, *Biosens Bioelectron* **2016**, *81*, 373-381.
